# Endogenous Opioid-Masked Latent Pain Sensitization: Studies from Mouse to Human

**DOI:** 10.1371/journal.pone.0134441

**Published:** 2015-08-25

**Authors:** Manuel P. Pereira, Renee R. Donahue, Jørgen B. Dahl, Marianne Werner, Bradley K. Taylor, Mads U. Werner

**Affiliations:** 1 Department of Anaesthesia, Centre of Head and Orthopaedics, Rigshospitalet, Copenhagen, Denmark; 2 Department of Physiology, University of Kentucky, Lexington, KY, United States of America; 3 Department of Cardiology, Rigshospitalet, Copenhagen, Denmark; 4 Multidisciplinary Pain Center, Neuroscience Center, Rigshospitalet, Copenhagen, Denmark; Nagoya University Graduate School of Medicine, JAPAN

## Abstract

Following the resolution of a severe inflammatory injury in rodents, administration of mu-opioid receptor inverse agonists leads to reinstatement of pain hypersensitivity. The mechanisms underlying this form of latent pain sensitization (LS) likely contribute to the development of chronic pain, but LS has not yet been demonstrated in humans. Using a C57BL/6 mouse model of cutaneous mild heat injury (MHI) we demonstrated a dose-dependent reinstatement of pain sensitization, assessed as primary (*P* < 0.001) and secondary hyperalgesia (*P* < 0.001) by naloxone (0.3–10 mg/kg), 168 hrs after the induction of MHI. Forward-translating the dose data to a human MHI model (n = 12) we could show that LS does indeed occur after naloxone 2 mg/kg, 168 hrs after a MHI. Our previous unsuccessful efforts to demonstrate unmasking of LS in humans are thus likely explained by an insufficient naloxone dose (0.021 mg/kg). However, while LS was consistently demonstrated in 21/24 mice, LS was only seen in 4/12 subjects. This difference is likely due to selection bias since the C57BL/6 mouse strain exhibits markedly enhanced pain sensitivity in assays of acute thermal nociception. Future exploratory studies in humans should prioritize inclusion of “high-sensitizers” prone to develop LS and use post-surgical models to elucidate markers of vulnerability to chronic postsurgical pain.

**Trial Registration:**

EudraCT 2012-005663-27

## Introduction

Injury-related nociceptive input to the CNS may trigger a sustained excitability and increased synaptic efficiency in central neurons [1], i.e., central sensitization, a stimulus-response enhancing mode which may contribute to the development and maintenance of chronic pain states [1,2]. Less appreciated, however, are data suggesting that central sensitization outlasts overt signs of hyperalgesia in a silent form termed “latent sensitization” (LS). We recently reported that constitutive activity at spinal mu opioid receptors maintains LS in a state of pain remission, but LS can be unmasked by administration of a centrally acting mu-opioid-receptor (MOR) inverse agonist, leading to “rekindling” or reinstatement of hyperalgesia [3]. Thus, LS could prime central nociceptive circuitry such that, when inhibitory systems fail, as upon exposure to excessive stress, a pain episode ensues [1,4,5]. This latent predisposition to relapse may explain the episodic nature and vulnerability to stressors that accompany chronic pain states in humans [1,4]. However, LS has not yet been reported in humans.

The objectives of the present study were therefore *first*, to delineate a naloxone dose-response curve for reinstatement of hyperalgesia in a preclinical mouse model of mild heat injury (MHI), and then, *second*, to forward-translate the results to a human MHI-model.

## Materials and Methods

### Study Approvals, Animals and Subjects

#### Approvals

The protocols were approved by the Institutional Animal Care and Use Committee (IACUC 2008–0337) of the University of Kentucky, and, the Capital Region’s Committee on Health Research Ethics (no.: H-2-2012-174), the Danish Health and Medicines Authority, the European Medicines Agency (mutual protocol number: EudraCT 2012-005663-27 [https://www.clinicaltrialsregister.eu/ctr-search/trial/2012-005663-27/DK]) and the Danish Data Protection Agency. The protocol and the CONSORT checklist are available as supporting information (**S1 Protocol**; **S1 CONSORT Checklist** **).**

#### Animals

Male C57BL/6 mice (Charles River Laboratories, Indianapolis, USA [n = 37]) were used.

#### Humans

**Fig 1:**
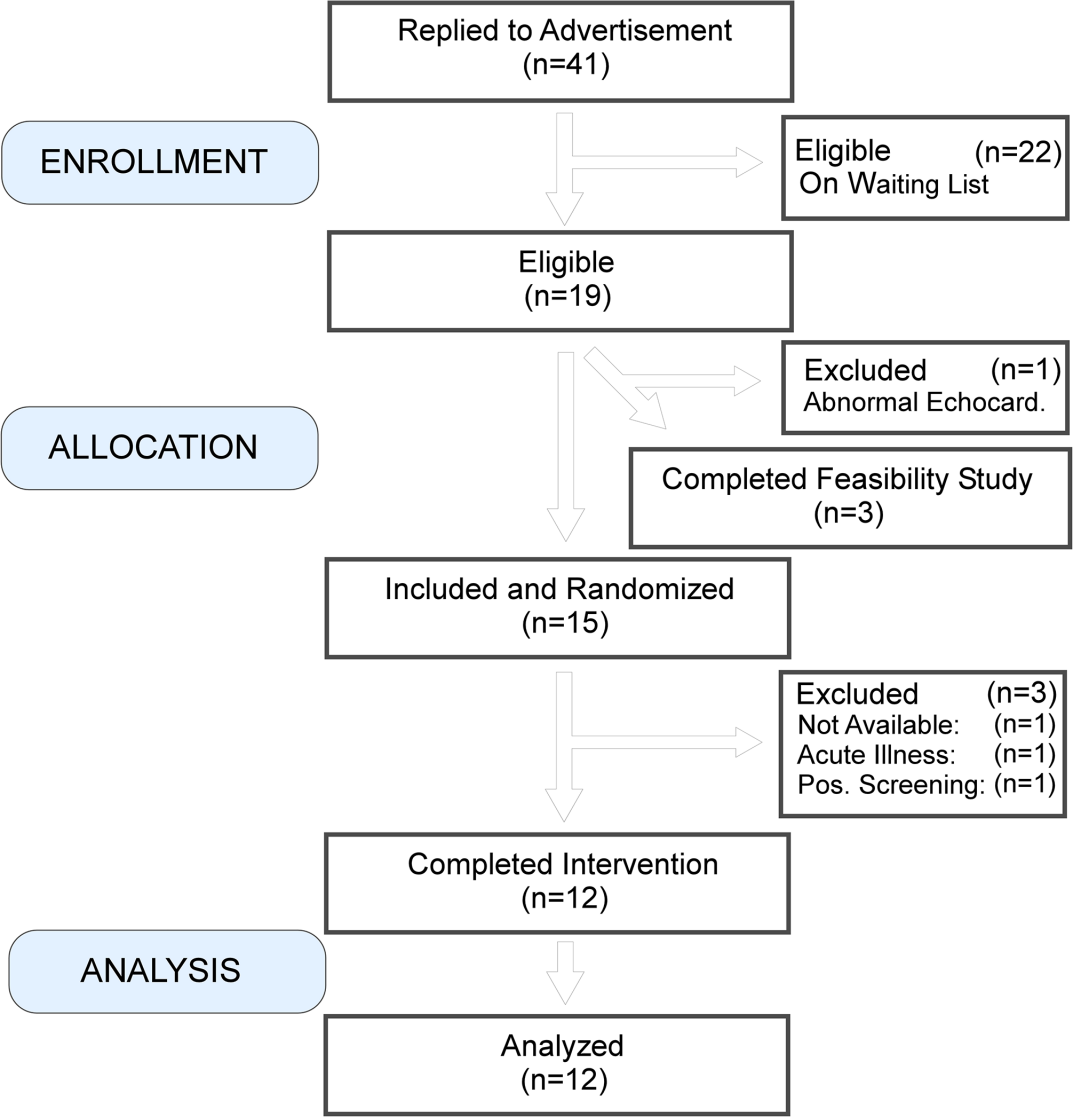
CONSORT flow-algorithm for human subjects. Four subjects were allocated to a feasibility study. One subject was excluded from this part due to an unsuspected finding of an abnormal ultrasound cardiography. Fifteen subjects were included and randomized. Three subjects were excluded for reasons not related to the study. Twelve subjects completed the study and data were examined in an interim analysis by an independent statistician.

Healthy male subjects were recruited from a registry of completed studies and by a website www.forsoegsperson.dk (inclusion and exclusion criteria Table 1). All subjects signed informed consent prior to participating in the study. Four pilot subjects were included to test the feasibility of the study. One subject was excluded due to abnormal findings in the echocardiography examination. No data from these pilots were included in the study material. Fifteen healthy males were included in the study on an intention-to-treat basis (Fig 1). The complete date range for participant recruitment and follow-up was June 3^rd^ to November 20^th^ 2013.

**Table 1:** Inclusion and exclusion criteria.

Inclusion Criteria	Exclusion Criteria
**- Healthy male**	- Volunteer is not cooperative
**- 20 ≤ age ≤ 35 years**	- Volunteer does not understand Danish
**- Urine sampled negative for amphetamines, barbiturates, benzo-diazepines, cocaine, opioids (bu-prenorphine, methadone, morphine) and tetra-hydrocannabinol (THC)**	- Participation in a drug trial in previous 60 days
**- 18 kg/m** ^**2**^ **< BMI < 30 kg/m** ^**2**^	- Regular use of analgesics (≥ twice a week)
**- Signed informed consent**	- Alcohol or drug abuse
**- Normal echocardiography**	- Use of psycho-active drugs
**- Normal ECG**	- Use of prescription drugs 1 week before study
	- Use of OTC- medication 48 hours before study
	- Allergy to opioids including naloxone
	- Chronic pain condition
	- Neurological or psychiatric disease
	- Skin lesions in the assessment areas

#### Ethical considerations

Reinstatement of hyperalgesia may require 2 mg/kg of naloxone parenterally, a dose 300 to 3,000 times higher than the dose recommended in humans, i.e., 0.04 to 0.4 mg per 70 kg BW, used in clinical treatment of an opioid overdose [6]. However, excessively high doses of up to 6 mg/kg of naloxone parenterally have been used in clinical [7,8] and experimental studies [9,10] with no or few adverse effects reported. A routine echocardiographic examination was made in all subjects in order to exclude cardiac abnormalities.

### Study Design

#### Animal study

The study was a randomized, observer-blind, vehicle-controlled, and five-arm parallel study.

#### Human study

**Fig 2:**
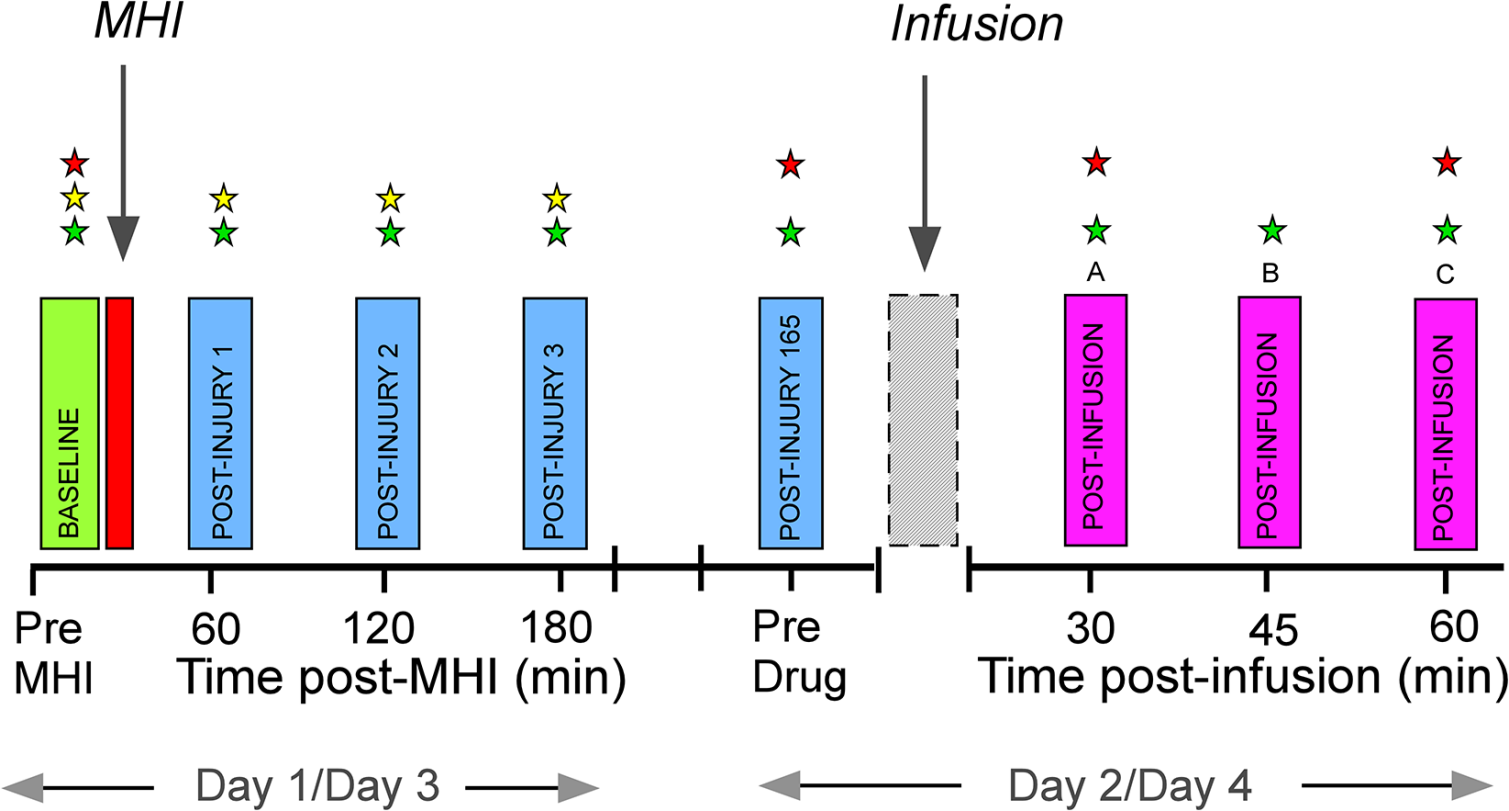
Study timeline in the human MHI-model. Day 1/Day 3 include induction of mild heat injury (MHI) with baseline assessments (green rectangle), heat injury (red) and post-injury assessments (blue). Day 2/Day 4 include a pre-drug assessment (blue; post-injury 165 hrs), drug-infusions (naloxone or placebo; grey) and post-infusion assessments (magenta). Stars indicate assessments of mechanical pain thresholds (red star), thermal thresholds (yellow) and secondary hyperalgesia areas (green).

The study was a randomized, placebo-controlled, double-blind, crossover study and was performed on 5 separate days (Fig 2). Day 0 was an inclusion assessment day with training of the quantitative sensory testing (QST) technique and the MHI. The MHIs were induced on Day 1/Day 3, and separated by 7 days from the drug administration days, Day 2 /Day 4, respectively. The washout period between the drug administration days was 40 (31 to 50) days.

The randomization procedure was performed by the Skanderborg Pharmacy using www.randomization.com. Block randomization with block sizes of 6 subjects was used. Naloxone (4 mg/ml) and normal saline were packed in identical 50 ml vials, and, packaging and the labelling were performed by the pharmacy. Subjects and investigators were blinded to the treatment sequence throughout the study. For each subject, two sealed non-transparent envelopes contained the randomization code. One of the envelopes was placed in the Trial Master File of the study, while the second was placed in the laboratory where the study took place, to be un-blinded in case of a medical emergency.

The *primary outcome* was MHI-related changes in secondary hyperalgesia areas (SHA) induced by naloxone Day 3/Day 4. The *secondary outcome* was brief heat sensitization (BHS) related changes in SHA induced by naloxone Day 3/Day 4.

### Environment

#### Animal study

All mice were received at 20 wks of age, housed two to four to a cage on a 14:10-hour light/dark cycle (6 am/8 pm), given food and water *ad libitum*, and acclimated to our facility (Department of Physiology, University of Kentucky, Lexington, KY) approximately one week before experimentation. Animals were acclimated to acrylic glass chambers, which rested on a wire mesh.

#### Human study

The experimental procedures were performed in quiet, well-lit laboratories (temperature: mean ± CI = 24.0 ± 0.5°C; relative humidity (RH) = 39 ± 2%) at the Multidisciplinary Pain Center and the Department of Anaesthesiology, Rigshospitalet, Copenhagen University Hospital. The testing sessions were done June 16^th^ to November 13^th^ 2013. Subjects adopted a comfortable supine position during the assessments and were allowed to move freely in adjacent rooms between the assessments.

### Mild Heat Injury and Brief Heat Sensitization

#### Animal study

The MHI-method was originally developed in the rat by Nozaki-Taguchi & Yaksh [11], and we adapted it for use in mice. In pilot studies we adjusted hotplate temperature and time of exposure to mimic the degree of injury produced in our human subject protocol: avoid blistering or swelling of the skin, yet produce heat hypersensitivity at the injured area (primary hyperalgesia) as well as tactile hypersensitivity at the undamaged tissue surrounding the injury (secondary hyperalgesia). Under isoflurane anesthesia (Butler Schein, USA) MHI was induced by placing the heel of the ventral surface of the left hindpaw on a 52.0°C hot plate for 40 seconds. Downward pressure was gently applied to the dorsal hindpaw to maintain constant contact between the heel and the hot plate.

#### Human study

The *MHI procedure* has been validated and previously described in a number of pharmacological and physiological studies [12–14]. MHI was induced at the medial aspect of the lower leg by a contact thermode with an active surface of 12.5 cm^2^ applied with gentle constant pressure maintaining 47.0°C for 420 seconds. All subjects developed localized erythema and hyperalgesia following the MHI, corresponding to a first degree burn injury. In order to avoid any between-day carry-over effect of the MHI [13] the non-dominant lower leg was used Day 1 and the dominant lower leg was used Day 3.

The *BHS* is a sensitization procedure using a short lasting, noxious heating of the skin without causing an injury. The SHA are measured after 3 min with the heated thermode *in situ*. The total testing time does not exceed 5 min. The BHS was used in order to test if naloxone had an effect on hypersensitivity in skin *not* previously injured, measured as pain perception and secondary hyperalgesia during continued heating. In *humans*, an area of 2.5 x 5.0 cm^2^, corresponding to the active surface of the thermode, was delineated on the anterior aspect of the thigh, with the lower border of the rectangle 11 cm superior to the upper border of the knee cap. A tonic heat stimulus (3 min, 45.0°C) was delivered to the dominant thigh (Days 1/Day 2) or non-dominant thigh (Days 3/Day 4), as previously described [13].

### Behavioral Testing

#### Animal study

In *mice* mechanical hypersensitivity was evaluated before and after MHI. Measurements were taken at the heel (site of heat application, to test for primary hyperalgesia) and between the tori (to test for secondary hyperalgesia). Each measurement of paw withdrawal threshold (PWT) started with the perpendicular application of the 0.16 g (1.57 mN) monofilament at the plantar hindpaw skin with sufficient force to cause a slight bending of the filament. If the animal did not respond, the next largest force was applied. An incremental series of 8 polyamide monofilaments of logarithmically increasing stiffness (0.08–58.86 mN = 0.008–6.0 g) were used. If a withdrawal response was noted, then the next smallest force was applied. This was repeated until either there was a response to all monofilaments, no response to the largest gram force used, or until four stimuli were applied after the initial change in response. The PWT_50%_ was thus determined using a modified Dixon’s up-and-down method [15,16].

#### Human study

Pain ratings during MHI and BHS: subjects rated the pain intensity during the MHI with a visual analogue scale (VAS; 0 = no pain, 100 = maximum imaginable pain) at 0, 30, 60, 120, 180, 240, 300, 360 and 420 s after the thermode had reached 47.0°C. In the BHS, subjects rated the pain intensity by VAS at 0, 30, 60, 120 and 180 s after the thermode had reached 45.0°C.

Assessment of SHA: the SHA were determined by stimulating with a polyamide monofilament (bending force 937 mN; Stoelting, IL, USA) along 8 diagonal and orthogonal lines converging towards the center of the MHI area or the *in situ* thermode in BHS. The subjects, who had their eyes closed during the measurements, reported the occurrence of a burning or stinging sensation as the stimulus was moved from normal skin into hyperalgesic skin. The corners of the octagon were marked on the skin and copied to a transparent sheet. Tactile allodynia areas were similarly assessed by stroking with a cotton wool bud. The secondary hyperalgesia and allodynia areas were calculated using a computer-based vector-algorithm (Canvas 12.0, ACD Systems International, Victoria, Canada). Measurements of SHA were performed Day 1/Day 3 (baseline, and, 1, 2, and 3 hrs post-MHI) and Day 2/Day 4 (before, 30 and 60 min post-infusion; Fig 2). Maxima of SHA were compared, i.e., post-infusion *vs*. pre-infusion values.

Assessment of mechanical pain thresholds: the MPT were assessed in the MHI area and the subjects were asked to indicate when at least three of the five perpendicularly applied pin-prick stimuli (“weighted-pin” stimulators: 8–512 mN; tip-area 0.049 mm^2^; PinPrick, MRC Systems, Heidelberg, Germany) were perceived as painful. Using pin-prick stimulators of ascending or descending order and using the method-of-limits algorithm, the MPT was determined five times and the median of these assessments was then considered for analysis. MPT assessments were performed at all study days (Day 1/Day 3: at baseline; Day 2/Day 4: before (165 hrs), and, after the infusion (168 hrs 07 min, and 168 hrs 37 min, post-MHI; Fig 2). Mean values of post-infusion MPT assessments were compared to pre-infusion MPT.

Assessment of thermal thresholds: *warmth detection threshold* (WDT) and *heat pain threshold* (HPT), were measured from a baseline temperature of 32°C with a ramp rate of 1.0°C/s and 50.0°C as the cut-off temperature. The assessments were each made three times and the median values were used in further analyses. Thermal thresholds were assessed Day 1 and 3, before and 1, 2 and 3 hours after MHI, respectively (Fig 2). Mean values of post-MHI thermal thresholds assessments were used for comparison with pre-MHI values.

Psychometrics:the subjects completed the Hospital Anxiety and Depression Scale (HADS) [17,18] and the Pain Catastrophizing Scale (PCS) [19] questionnaires.

### Experimental Timeline and Drug Administration

#### Animal study

Seven days after induction of MHI, either saline vehicle (n = 7) or naloxone HCl (Tocris, MN, USA; 0.03 [n = 6], 0.3 [n = 8], 3.0 [n = 8] or 10.0 [n = 8] mg/kg, i.p.) was injected and mechanical sensitivity was reassessed post-injection. Each animal was injected twice using a cross-over design, with 7 days separating the first injection of drug or vehicle from the second injection of vehicle or drug, respectively. For any given treatment, mechanical thresholds following the first and second injections did not differ, and so were combined for graphical presentation and statistical analysis.

#### Human study

Seven days after induction of MHI (Day 2/Day 4; Fig 2), subjects received a 17 min infusion of naloxone (2 mg/kg) or normal saline. Drug administration was an i.v. test-bolus of 1 ml (4 mg of naloxone or normal saline) administered during 2 minutes, followed by a weight-based infusion delivering a total volume of naloxone 0.5 ml/kg (2 mg/kg) or normal saline 0.5 ml/kg in 16 min 40 s. Measurements on Day 2/Day 4 were carried out before and after the infusion (Fig 2).

### Statistics

#### Sample size estimation

In the human part of the study the *a priori* sample size calculation was based on estimates of an intra-individual standard deviation (SD) of secondary hyperalgesia areas of 3 cm^2^, a minimal relevant difference of 3 cm^2^, a significance level of 0.01 (α) and a power of 0.90 (β = 0.10). These data were extrapolations based on assumptions from our previous negative low-dose naloxone study [13]. Assuming that data were normally distributed, utilizing a cross-over design and an effect size of 0.71, the estimated number of subjects needed were 34 (G*Power version 3.1.7, University of Kiel, Germany). The study was prematurely discontinued after inclusion of 6 subjects, since an *a priori* planned interim, futility analysis, by an independent, blinded statistician estimated that the number needed to show a statistical difference between the treatments was at least 187 subjects (α = 0.01, β = 0.10, effect size = 0.31, Wilcoxon matched pairs test, distribution: A.R.E. [asymptotic relative efficiency] method). No superiority analysis was performed. However, since 6 additional subjects at this time point were near completion of the study, due to the exploratory nature of the study, it was decided that data from the 6 additional subjects would be included in a final *post hoc* analysis. The investigators were kept unaware of the result of the *a priori* planned interim analysis until after the last subject’s last study visit. The *post hoc* analysis, thus including the 12 subjects, indicated at least 66 subjects would be needed to demonstrate a statistical difference between treatments (α = 0.01, β = 0.10, effect size = 0.53, Wilcoxon matched pairs test, distribution: A.R.E. method).

#### Statistical analyses

Analyses were carried out using SAS Institute software (9.3, Cary, NC), SPSS software (20.0, Chicago, IL) and MedCalc Software (12.3.0.0, Mariakerke, Belgium). Human data sets were initially assessed for normality by the Kolmogorov-Smirnov test and inspection of residual plots. Between-treatment effects were statistically compared using two-tailed t-tests or Wilcoxon matched pairs tests, as appropriate. For the *mouse* studies, we used a linear mixed model relating the outcome at each time point to the dose and time, controlling for baseline, and with random effects to capture the correlations among repeated measurements. Linear contrasts with Benjamini-Hochberg adjustments for multiple comparisons were used to test whether there were dose effects at each time point. For the *human* studies time-dependent, between-group changes in sensitivity, were evaluated by two-way analyses of variance (ANOVA) and repeated measures ANOVA, as appropriate. The level of significance for statistical comparisons was set to 0.01 in order to decrease the risk of type I error due to multiple comparisons. Data are presented as mean or median (95% CI) and in small samples median (25–75% interquartile range [IQR]).

## Results

### Exclusion

A total of 15 subjects were included with intention-to-treat in the study, however, 3 subjects were excluded for reasons not related to the study (Fig 1): one subject did not show up after the first visit, one subject had an acute illness on Day 2, not related to the study drug, and one subject tested positive for opioids on Day 4. Hence, per-protocol data from 12 subjects were included in the study (anthropometric data in Table 2).

**Table 2:** Anthropometric data (n = 12). BMI = body mass index.

	Age (yrs)	Height (cm)	Weight (kg)	BMI (kg/m^2^)
**Mean**	23.8	186.5	77.5	22.2
**(95% CI)**	(22.8–24.9)	(182.4–190.6)	(72.2–82.9)	(21.3–23.1)

### Behavioral Testing

#### Animal study

**Fig 3:**
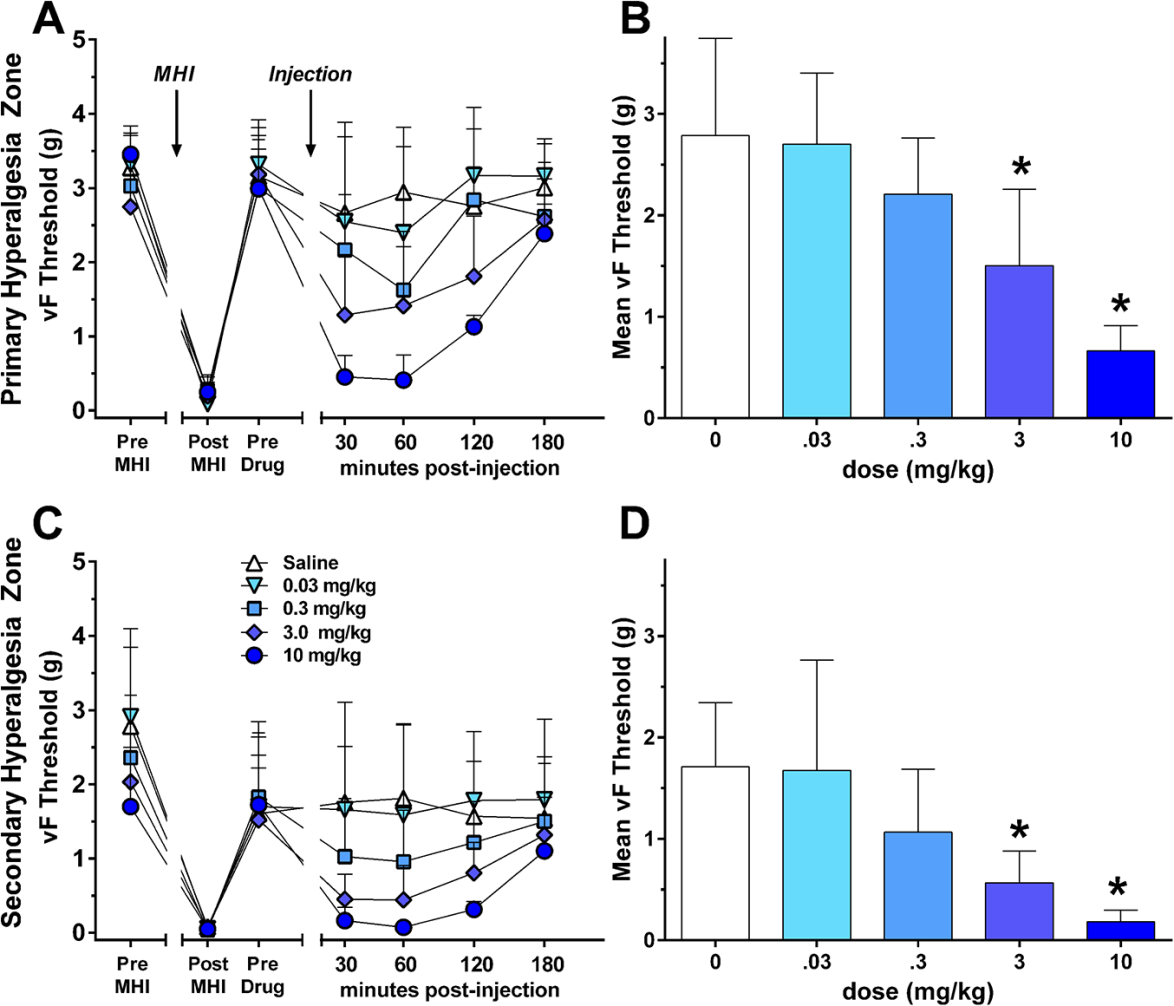
Hyperalgesia was assessed by a decrease in paw withdrawal threshold (PWT_50%_) in a model of mild heat injury (MHI). Line graphs illustrate response to von Frey (vF) stimulation at baseline (BL) and at 1, 2, 3, 72, and 168 hrs after MHI. Naloxone reinstated primary hyperalgesia (**A-B**) at a dose of 0.3 mg/kg (60 min, *P* = 0.0015), 3 mg/kg (30 min, *P* = 0.0011; 60 min, *P* = 0.0002), and 10 mg/kg (30 min, *P* < 0.0001; 60 min, *P* < 0.0001; 120 min, *P* < 0.0001) and secondary hyperalgesia (**C-D**) at a dose of 0.3 mg/kg (60 min, *P* = 0.0148), 3 mg/kg (30 min, *P* = 0.0004; 60 min, *P* = 0.0002), and 10 mg/kg (30 min, *P* < 0.0001; 60 min, *P* < 0.0001; 120 min, *P* = 0.0013). **A,C**: Line drawings. **B,D**: Histograms of the post-injection time points (plotted as the mean of the 30–120 min time points). Values represent mean ± 95% CI. * *P* < 0.05.

MHI rapidly (1 hr) produced primary and secondary hyperalgesia that gradually returned to pre-injury baseline values within 168 hrs (Fig 3). Naloxone dose-dependently reinstated both primary (*F*_(4,168)_ = 13.9, *P* < 0.001; Fig 3A and 3B) and secondary hyperalgesia (*F*_(4,168)_ = 6.1, *P* < 0.001; Fig 3C and 3D; **S1 Table**). The significant effect at 3 mg/kg informed the 2 mg/kg dose administered to humans.

#### Human study

**Fig 4:**
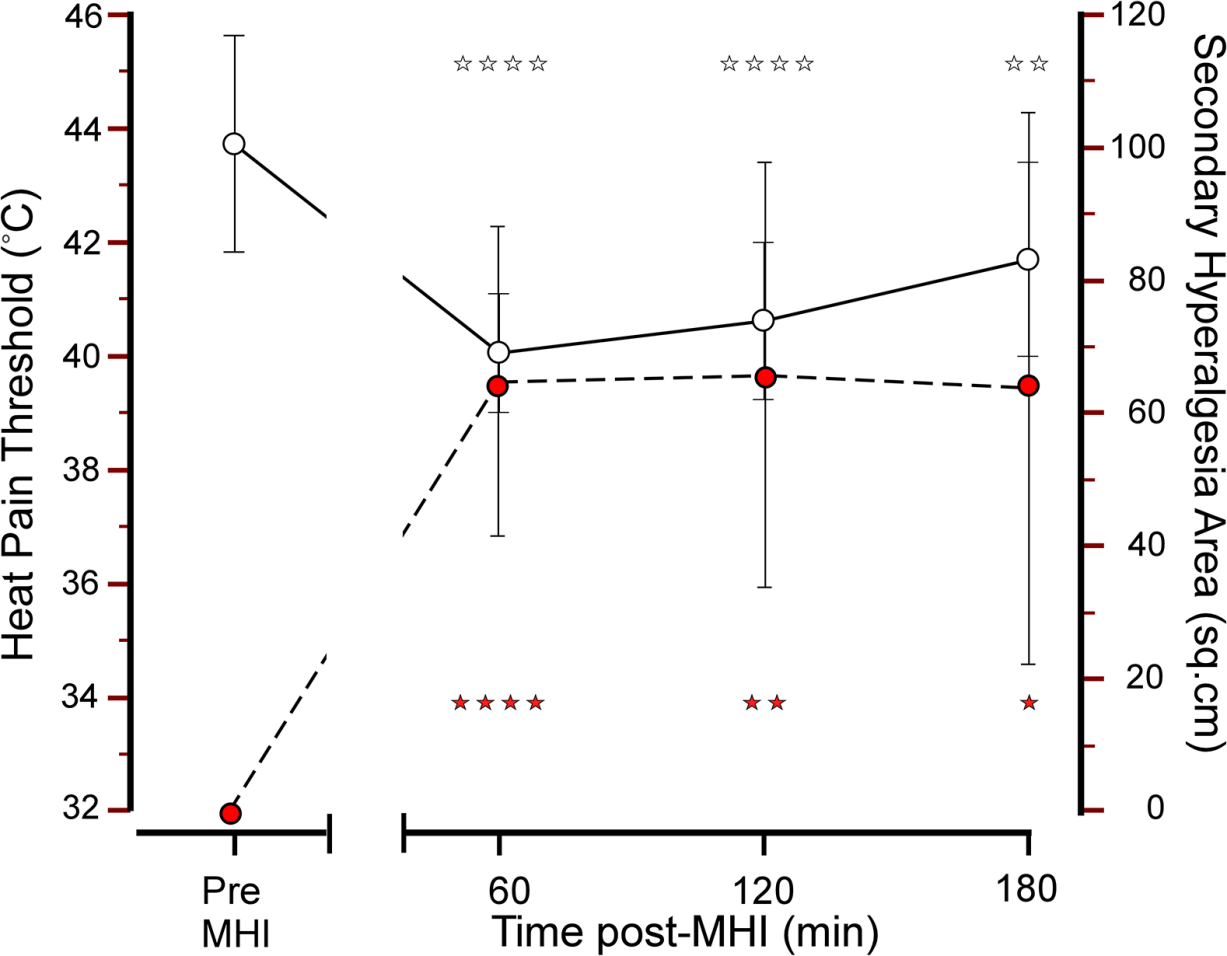
Heat pain thresholds and secondary hyperalgesia areas before and after a mild heat injury. Heat pain thresholds (white circles; left y-axis) and secondary hyperalgesia areas (red circles; right y-axis) before and, 60, 120 and 180 min, after a mild heat injury (MHI). Values represent mean of Day 1 and Day 3 values (mean ± 95% CI). * *P* < 0.01; ** *P* < 0.005; **** *P* < 0.0005.

*Changes induced by MHI Day 1/Day 3*: pain intensity was 32 (24–39) visual analogue scale units (VAS; 0–100) with no difference between Day 1 and Day 3. MHI produced a robust and short-lasting primary and secondary hyperalgesia as reflected by the development of heat (*F*_(3,24)_ = 26.1, *P* < 0.00001) and mechanical hypersensitivity (*F*_(3,33)_ = 13.5, *P* < 0.00001, Fig 4; **S2 Table**).

*Changes induced by BHS Day 1/Day 3*: all subjects developed SHA following the BHS challenge. Pain during the BHS-induction was 12 (6–18) VAS-units with no difference between Day 1 and Day 3 (*P* = 0.10), and no sign of sequence effect was observed (*P* = 0.58). The immediate post-MHI phase demonstrated significant development of SHA (*F*_(4,44)_ = 7.6, *P* = 0.0001; **S2 Table**). A significant carry-over effect between Day 1 and Day 3 (*P* = 0.0049; Day 1 > Day 3) was seen, but there was no sign of a drug-sequence effect (*P* = 0.47).

**Fig 5:**
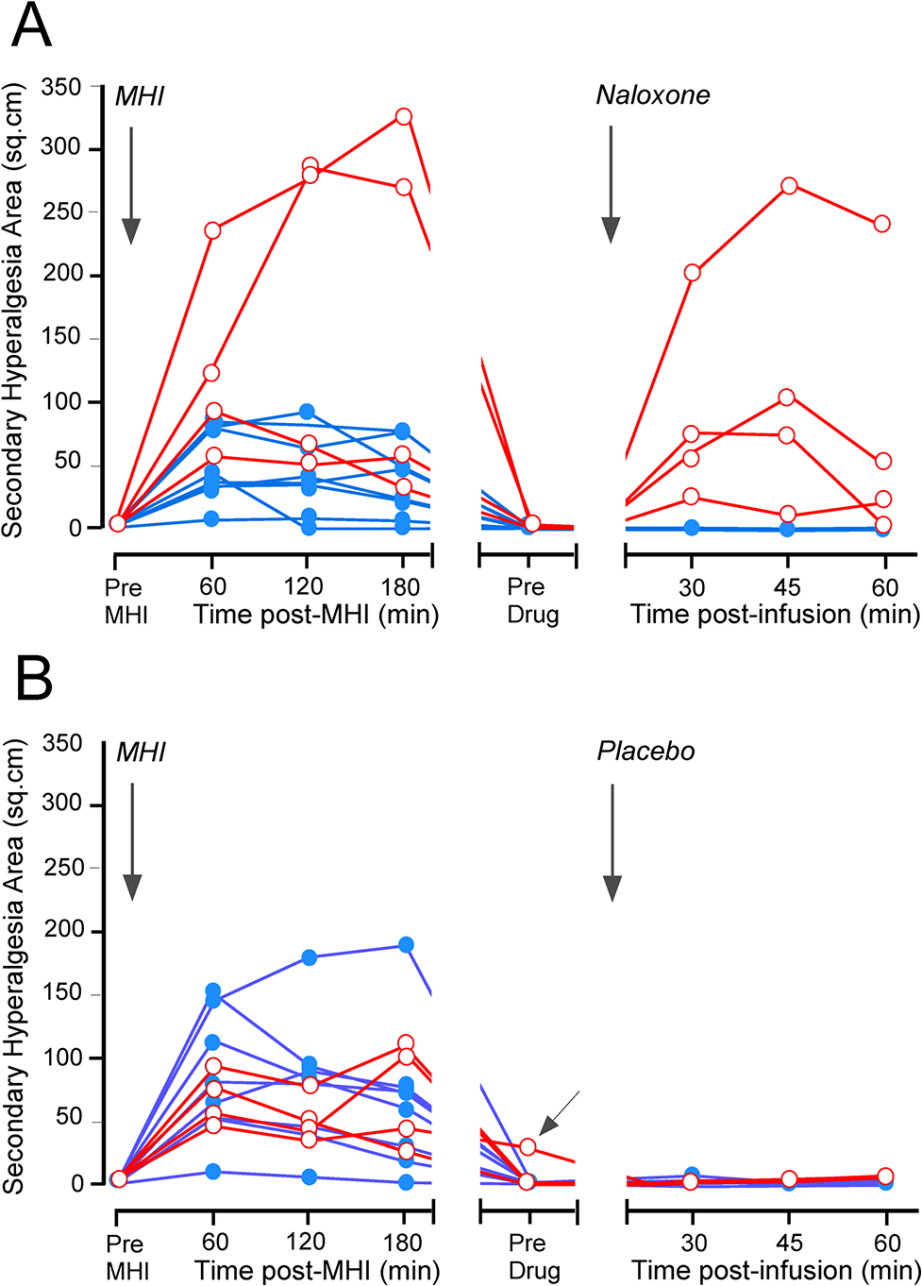
Individual trajectories of secondary hyperalgesia areas during the early and late phase after a mild heat injury. Individual trajectories of secondary hyperalgesia areas (n = 12) during the early phase (0–3 hrs) and the late phase (165–169 hrs) after induction of the mild heat injury (MHI). Data are from naloxone (**A**) and placebo (**B**) sessions. The trajectories of the four naloxone responders, indicating presence of endogenous opioid masked sensitization, are delineated by red circles and the trajectories from the eight non-responders by blue filled circles. MHI and drug administration are indicated by vertical arrows. One (#11) of the responders demonstrated residual SHA at 165 hrs after the induction of MHI (indicated by oblique arrow; **B**), but not during placebo. Secondary hyperalgesia areas developed in 4/12 subjects (#2,6,7,11) after naloxone (**A**) and in 0/12 after placebo (**B**).

*MHI-related changes in SHA induced by naloxone Day 3/Day 4*: after recovery of hyperalgesia indices at 168 hrs after MHI, the naloxone infusion produced large secondary hyperalgesia areas (SHA) in 4/12 subjects (median [25–75% IQR]: 90.3 cm^2^ [63.2–145.3 cm^2^]; #2,6,7,11; Fig 5A), while the placebo infusion did not produce SHA in any subject (0 cm^2^ [0–1 cm^2^]; Fig 5B; **S2 Table**). Two subjects on the saline day and one subject on the naloxone day had residual secondary hyperalgesia areas (SHA) 165 hrs post-MHI: #9 (1.4 cm^2^) and #11 (28.9 cm^2^), and, #7 (2.6 cm^2^), respectively.

*BHS-related changes in SHA induced by naloxone Day 3/Day 4*: following naloxone-infusion 4/12 subjects developed large SHA (the area after placebo-infusion subtracted: median [25–75% IQR]: 83.8 cm^2^ [61.2–111.3 cm^2^]; #2,6,7,11; **S2 Table**), while in the remaining 8/12 the SHA were much smaller (0 cm^2^ [-5.1–5.0 cm^2^]). *Interestingly*, these responder-subjects were the same subjects that also developed the largest SHA following the MHI.

*Psychometrics*: none of the subjects scored values outside the normal range in the HADS or PCS questionnaires (**S2 Table**).

### Adverse Drug Related Effects

Six study subjects reported mild side effects after administration of naloxone: 3 subjects experienced mild to moderate tiredness, 2 subjects experienced a frontal headache and 1 subject experienced epigastric pain. In addition, 1 subject reported intense photophobia, which lasted until the following day.

## Discussion

Administration of MOR inverse agonists to mice following resolution of the pain associated with plantar incision or cutaneous inflammation, is associated with *N*-methyl-D-aspartate receptor (NMDAR) dependent reinstatement of mechanical hypersensitivity at the injury site [3,20,21]. Our current findings with the inverse agonist naloxone, indicate for the first time that cutaneous MHI triggers a state of LS not only in mice, but remarkably also in humans. Previous unsuccessful efforts to demonstrate naloxone-dependent unmasking of LS in humans [13] are likely explained by insufficient dosing (0.021 mg/kg). As we recently described in animal studies [3], injury induces constitutive MOR activity that can prevent the transition from acute to chronic pain. As chronic pain is a well-recognized major medical problem [22,23] our results may have profound implications in pain research and management.

### “High Sensitizers”?

Is there a subgroup of humans who are predisposed to LS? We believe that there could be: the initial SHA (median [25–75% IQR] 1–3 hr after MHI) were much higher in the 4/12 subjects showing LS (144.4 cm^2^ [60.3–240.3 cm^2^]; Fig 5) as compared to the 8/12 subjects not showing LS (38.7 cm^2^ [23.6–74.0 cm^2^]). Thus, subjects presenting with large initial SHA also showed naloxone-dependent hyperalgesia reinstatement. This raises the possibility that “high-sensitizers” [14] are more prone to development of LS, and is consistent with reports indicating that level of pain before surgery is a key predictor of the development of chronic pain after surgery [24,25]. In contrast to the human studies nearly all the mice (8/8 at 10 mg/kg, 7/8 at 3 mg/kg, 6/8 at 0.3 mg/kg, 3/6 at 0.03 mg/kg, 2/7 at 0.0 mg/kg) exhibited pain reinstatement upon naloxone challenge (50% or more decrease in latency within one hour of injection). This difference is likely due to selection bias: genetic studies indicate that the inbred C57BL/6 mouse strain (used in the present study) exhibits markedly enhanced pain sensitivity in assays of acute thermal nociception as compared to other strains [26,27] and thus can be considered belonging to a “high-sensitizer” clone [26,28]. Irrespective of species difference, our results set the stage both for clinical trials that will select subjects with a propensity for developing central sensitization (“high-sensitizers”) and for surgical studies to elucidate markers of vulnerability to chronic postsurgical pain.

### Contralateral Changes

The 4/12 subjects who developed naloxone-dependent SHA *also* exhibited increased SHA during brief heat sensitization (BHS) challenge at the contralateral extremity. This is consistent with our recent report demonstrating naltrexone-induced robust hyperalgesia and spinal cord neuronal activation at the side contralateral to injury [3]. Mirror-image sensory dysfunction, i.e., *tertiary* hyperalgesia [1], has been described in persistent post-surgical pain, diabetic polyneuropathy and postherpetic neuralgia [29].

### Limitations

An important limitation is that following a planned interim-analysis the study was discontinued due to the large number of subjects (n ≥ 66) needed to demonstrate LS. As mentioned above the effect size in future studies may be increased by selecting “high sensitizers” (subjects whom develop large SHA) or by studying surgical models with a greater volume of tissue damage, thus enhancing central sensitization drive.

The dose of naloxone necessary to un-mask LS is hundred to thousand times higher than the doses used to reverse severe opioid overdose in humans. Unpublished data from a positron emission tomography (PET) study in humans demonstrated complete inhibition of [^11^C]-carfentanil binding to opioid-receptors following 100 microg/kg naloxone [30]. We speculate that greater doses are needed to sufficiently block MOR constitutive activity [3], but cannot rule out actions of high dose naloxone at unknown “off-target” receptors.

## Conclusion

In conclusion, our data suggest that LS occurs in humans, and provide proof of principle for large-scale clinical studies to facilitate our understanding of the mechanisms involved in the transition from acute to chronic pain, and to begin to elucidate markers of LS and thus vulnerability to chronic pain, e.g., by assessing the hyperalgesic response to naloxone following surgery.

## Supporting Information

S1 CONSORT Checklist Consort 2010 Checklist (PDF-file). (PDF)

S1 Protocol Protocol (PDF-file). (PDF)

S1 Table Mild Thermal Injury Animal Data. Table with individual dose-response data on primary and secondary hyperalgesia assessed by monofilaments (von Frey hair) before and after a MHI (Excel-file).
(XLSX)

S2 Table Mild Thermal Injury Human Data. Table with individual data on demographics, laboratory environment (temperature and relative humidity), psychometrics (HADS and PCS), VAS pain scores, thermal thresholds (WDT and HPT), mechanical pain thresholds, MHI-related secondary hyperalgesia and allodynia areas and BHS-related secondary hyperalgesia and allodynia areas (Excel-file).
(XLSX)
