# Supplementary material for: Endogenous Opioid-Masked Latent Pain Sensitization: Studies from Mouse to Human
Source: PLoS One. 2015 Aug 25;10(8):e0134441. doi: 10.1371/journal.pone.0134441 (PMC4549112; doi:10.1371/journal.pone.0134441)
Supplement: S1 Protocol — (PDF) [file pone.0134441.s002.pdf]

PROTOKOL

Effekten af sen høj-dosis naloxon-infusion på sekundær hyperalgesi efter en førstegrads varmeskade

Sponsor:
Navn: Jørgen B. Dahl
Titel: professor, overlæge, dr.med

Ansvarlig investigator:
Navn: Mads U. Werner
Tiltel: overlæge, med. dr.

Subinvestigatorer:
Navn: Thomas Ringsted
Titel: Forskningssygeplejerske

Navn: Manuel P. Pereria
Titel: Læge

Navn: Joakim M. Bischoff
Titel: Klinisk assistent

Protokol, Version nr.5-1 amendment 1,
dato 22-02-2013
Protokol nr. MWTR-5

Diverse navne, adresser, tlf nr. samt e-mailadresser af involverede i studiet.

Sponsor:

Jørgen B. Dahl, professor, overlæge,
dr.med. Københavns Universitet
Anæstesiologisk afd. 4231, HOC
Rigshospitalet, Blegdamsvej 9, 2100
København Ø Tlf.: 3545 3475
E-mail: joergen.b.dahl@rh.regionh.dk

Principal investigator:

Mads U. Werner, overlæge, med. dr.
Tværfagligt Smertecenter, afsnit 7612, Neurocentret,
Rigshospitalet, Blegdamsvej 9, 2100 København Ø
tlf.: 28 25 77 03
E-mail: mads.u.werner@gmail.com

Sub- investigator:

Thomas Ringsted, projektsygeplejerske
Tværfagligt smertecenter, afsnit 7612, Neurocentret
Rigshospitalet, Blegdamsvej 9, 2100 København Ø
tlf.: 3545 9502
E-mail.: thomaskringsted@hotmail.com

Manuel P. Pereira, læge
Anæstesiologisk afd. 4231, HOC
Rigshospitalet, Blegdamsvej 9, 2100 København Ø
Tlf.: 3545 3475
E-mail.: mpflp47@gmail.com

Joakim M. Bischoff, læge, klinisk assistent
Tværfagligt smertecenter, afsnit 7612, Neurocentret
Rigshospitalet, Blegdamsvej 9, 2100 København Ø
tlf.: 3545 7618
E-mail: jomutahi@hotmail.com

Monitor:

Københavns Universitetshospitals GCP-enhed
Bispebjerg Hospital, Bygning 51, 3.sal
Bispebjerg Bakke 23
2400 København NV
Tlf. 35 31 38 90

Samarbejdspartnere:

Farmaceut Aage Rasmussen
Skanderborg Hospitals Apotek
Skanderborg Sundhedscenter
Sygehusvej 7
8860 Skanderborg
Tlf: 7842 9000
E-mail: ara@skanderborg-apotek.dk

Bradley Taylor
Professor, Dept. Physiology, MS-508
University of Kentucky Medical Center
800 Rose Street
Lexington, KY 40536-0298
brad.taylor@uky.edu

Effekten af sen høj-dosis naloxon-infusion på sekundær hyperalgesi efter en førstegrads varmeskade

17-12-2012

Undertegnede sponsor og principal investigator bekræfter hermed til enhver tid at følge protokollens ordlyd og arbejde efter de gældende regler for Good Clinical Practice.

Sponsors navn og adresse:
Jørgen B. Dahl, professor, overlæge, dr.med.
Københavns Universitet
Anæstesiologisk afd. 4231, HOC
Rigshospitalet

Sponsors dato og signatur: 17/12-2012 [signature]

Principal investigators navn og adresse:
Overlæge Mads Werner
Tværfagligt Smertecenter, afsnit 7612, Neurocentret,
Rigshospitalet, Blegdamsvej 9, 2100 København Ø

Principal investigators signatur og dato: 19/12-2012 / Mads Werner

Indholdsfortegnelse

PROTOKOLTITEL 1

DIVERSE NAVNE OG ADRESSER PÅ INVOLVEREDE I STUDIET 2

SIGNATURSIDE 4

INDHOLDSFORTEGNELSE 5

INTRODUKTION OG RATIONALE 7
- BAGGRUND 7
- HYPOTESE 8
- LITTERATURLISTE 9

FORMÅL 11
ENDPOINTS 11
- PRIMÆRE ENDPOINTS 11
- SEKUNDÆRE ENDPOINTS 11

ETISKE OVERVEJELSER 11
- OVERVEJELSER I FORBINDELSE MED AFPRØVNINGEN SOM HELHED 11
- RISICI, ULEMPER MV. 12
- INFORMATION AF OG SAMTYKKE FRA PATIENTER 14
- BESKYTTELSE AF DATA FRA PATIENTER 15

RAMMER FOR STUDIET 16
- TIDSPLAN 16
- STED FOR UNDERSØGELSENS UDFØRELSE 16
- STUDIEDESIGN 17

STUDIESELEKTION 17
- INKLUSIONSKRITERIER 17
- EKSKLUSIONSKRITERIER 17

PRAKTISKE FORHOLD 18
- ØKONOMISKE FORHOLD 18
- PATIENTERS GENNEMFØRLESE OG AFBRYDELSE AF FORSØGET 19
- ÅRSAGER TIL PATIENTERS AFBRYDELSE AF FORSØGET 19
- PROCEDURE FOR PATIENTER DER AFBRYDER FORSØGET 19

METODOLOGI 19
- GENEREL BEHANDLINGSPLAN OG MEDICINDOSERING 19
- PSYKOMETRISKE SKALAER 22
- TÆRSKELBESTEMMELSER 23
- VARMESKADEN 24
- KORT VARMESTIMULATION 24

MEDICIN OG MEDICINHÅNDTERING 24
- UNDERSØGELSESMEDICIN 24
- BLINDINGSPROCEDURE, PAKNING OG ETIKERING 25
- ANDEN BEHANDLING 25
- PROCEDURER VED NØDSTILFÆLDE 25
- MEDICINHÅNDTERING 26
- MEDICINREGNSKAB 26

BIVIRKNINGER 26
- BIVIRKNING/UØNSKET HÆNDELSER (AEs = ADVERSE EVENTS) 26
- RETNINGSLINIER FOR UØNSKEDE HÆNDELSERS 27
- GRADERING AF UØNSKEDEDE HÆNDELSER 28
- RAPPORTERING AF AE'S OG SAE'S BIVIRKNINGER 28

STATISTISKE ANALYSER 29
- BEREGNING AF ANTAL PATIENTER 29
- DATABEARBEJDNING 31

DATAREGISTRERING, SAMT REGLER FOR KONTROL AF UNDERSØGELSESPROCEDURER 31
- CASE REPORT FORMS 31
- MONITORERING 31
- UDDANNELSE 31

YDERLIGERE KRAV OG GENEREL INFORMATION 32
- FORSIKRING 32
- OFFENTLIGGØRELSE AF RESULTATER 32

BILAG 1: DELTAGERINFORMATION, DET VIDENSKABSETISKE KOMITÉSYSTEM, SAMTYKKE- OG FULDMAGTSERKLÆRING

BILAG 2: PROTOKOLRESUME

BILAG 3: IDENTIFIKATIONSLISTE

BILAG 4: CASE REPORT FORM

BILAG 5: SPØRGESKEMAER (PCS OG HADS)

BILAG 6: Litteratur-oversigt over bivirkninger ved administration af i.v. høj-dosis naloxon

BILAG 7: Annonce: rekruttering af frivillige forsøgspersoner.

Introduktion og rationale

Baggrund:

Naloxon er et interessant farmakologisk værktøj idet det har dosis-afhængige anti-nociceptive og pro-nociceptive egenskaber.

I en eksperimentel, human smertemodel med intradermal elektrisk stimulering observerede man signifikante øgninger i sekundære hyperalgesi-arealer efter i.v. infusion af naloxon (21 mikrog/kg over 30 minutter [infusion startet 1 time efter start af den elektriske stimulering]).[1] Lokal administration af naloxon i et område med varmeskade har vist opioid-antagonistiske effekter.[2;3] Fra en af disse undersøgelser er det vist at naloxon responsiviteten afhænger af den individuelle smertesensitivitet.[2] Undersøgelserne tyder på at det endogene opioide system har en central og perifer modulerende (tonisk?) effekt på hyperalgesi responset.

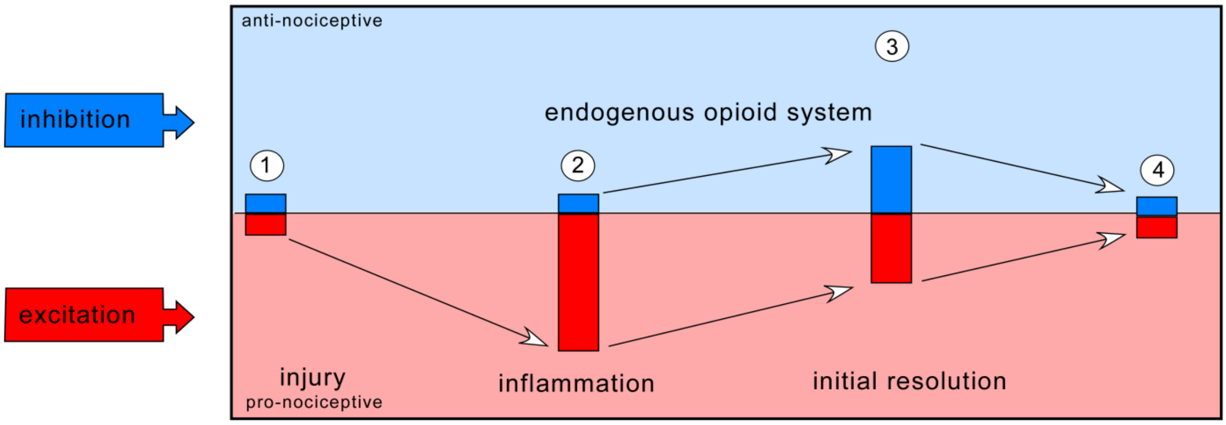

Figur 1. Det hypotetiske samspil mellem de inhibitoriske (anti-nociceptive [blå søjler]) og excitatoriske (pro-nociceptive [røde søjler]) systemer ved en vævsskade. Tallene angiver: (1) fysiologisk ækvilibrium, (2) skaden medfører en inflammatorisk reaktion, (3) lægningsfase med latent hyperalgesi (patologisk ækvilibrium), (4) fuld restitution med

overgang til fysiologisk ækvilibrium (idé efter Celerier et al.[4]) (Celerier et al. bruger en analog model til at forklare opioid-induceret hyperalgesi (OIH) og tolerance (OIT).

Upublicerede data (Brad Taylor November 2012, personlig kommunikation) fra dyre-eksperimentelle undersøgelser på mus viser at intraplantar injektion af ”complete Freund’s adjuvant” (CFA = BCG-vaccine) giver en nociceptiv hypersensitivitet i området som forsvinder efter 21døgn. Ved systemisk eller intrathekal tilførsel af naltrexon (analog til naloxon) efter døgn 21 ser man en re-introduktion af mekanisk og termal hypersensitivitet. Disse fund antyder at aktivitet i det endogene opioid system persisterer efter at de intiale tegn på hyperalgesi er forsvundet, og formentlig maskerer en latent nociceptiv sensitivering med fravær af smerte-lignende adfærdsændringer (fig. 1).

Hypotese:

Vores overordnede hypotese er at vi ved at bruge en varmeskademodel, som er kendt for at inducere en robust hyperalgesi, vil være i stand til, gennem naloxon-tilførsel 168 timer efter varmeskaden, at reintroducere sekundær hyperalgesi.

Varmeskademodellen er brugt indenfor farmakologiske,[5;6] neurofysiologiske[7] og prædiktive studier.[8;9] Modellen er valideret af forskellige forskningsgrupper.[10-12] Metoderne til induktion af varmeskaden og til måling omkring varmeskaden er tidligere godkendt i protokollerne (H-1-2009-132, H-2-2010-115, H-4-2011-101, H-2-2012-036).

Resultaterne af et identisk studie H-2-2012-036, med naloxon indgift, 21 mikrog/kg, der blev genemført juni-oktober i år, viser en paradoksal trend mod en mindskning af de sekundære hyperalgesi arealer efter indgift af naloxon sammenlignet med placebo, både i varmeskade modellen og i BTS (”Brief Thermal Sensitization”]). Vores formodede hypotese, at naloxon ville genfremkalde sekundære hyperalgesi arealer sammenlignet med placebo, er således forkastet, faktisk taler meget for at en modsat-rettet mekanisme er gældende: lav-dosis naloxon har en anti-hyperalgetisk effekt medens høj-dosis naloxon må formodes at have en hyperalgetisk effekt.

Vi har derfor nøje gennemgået de dyreeksperimentelle metoder og data (Bradley, personlig kommunikation), der tydeligt bekræfter vores oprindelige hypotese, og

sammenlignet dem med vores eget studie. Vi har fundet en del metodologiske forskelle, omkring tider og målinger, men den mest signifikante forskel er i dosis størrelse. Vi har derfor lavet en litteratur oversigt (Bilag 5) over høj-dosis naloxon indenfor human forskning. Oversigten, som indeholder 20 studier, viser at der er brugt intravenøse naloxon doser fra 2 – 4 mg/kg hos frivillige og fra 2 – 5 mg/kg hos patienter. Til patienter er der brugt intravenøse naloxon infusioner med indgift av 5 – 70 mg/kg indenfor 1 døgn. Sammenfattningsvis er de rapporterede bivirkninger dosis-relaterede, uden alvorligere bivirkninger selv hos kritisk syge patienter. Indenfor dosis-intervallet 2 til 4 mg/kg til frivillige forsøgspersoner er de beskrevne bivirkninger af mild intensitet og ser ud til at forekommer hos et mindretal: kvalme, opkast, svaghed, træthed, tremor og øgning af blodtryk og respirationsfrekvens.

Vi mener at kunne forsvare brugen af høj-dosis naloxon til raske forsøgspersoner, som inden undersøgelsen får foretaget en klinisk lægeundersøgelse og screenes med EKG og ultraludsundersøgelse af hjertet (UKG). Forsøgsdeltageren monitorereres før og efter naloxon administrationen med EKG, pulsoxymetri- og blodtryks-målinger. Undersøgelsen er vigtig for at kunne belyse mekanismer ved overgang mellem akut og langvarig smerte.

Litteraturliste

1. Koppert W, Filitz J, Troster A, Ihmsen H, Angst M, Flor H, Schuttler J, Schmelz M: Activation of naloxone-sensitive and -insensitive inhibitory systems in a human pain model. J.Pain 2005; 6: 757-64

2. Robertson LJ, Hammond GR, Drummond PD: The effect of subcutaneous naloxone on experimentally induced pain. J Pain 2008; 9: 79-87

3. Robertson LJ, Drummond PD, Hammond GR: Naloxone antagonizes the local antihyperalgesic effect of fentanyl in burnt skin of healthy humans. J Pain 2007; 8: 489-93

4. Celerier E, Laulin JP, Corcuff JB, Le MM, Simonnet G: Progressive enhancement of delayed hyperalgesia induced by repeated heroin administration: a sensitization process. J Neurosci. 2001; 21: 4074-80

5. Dirks J, Fredensborg BB, Christensen D, Fomsgaard JS, Flyger H, Dahl JB: A randomized study of the effects of single-dose gabapentin versus placebo on postoperative pain and morphine consumption after mastectomy. Anesthesiology 2002; 97: 560-4

6. Werner MU, Lassen B, Kehlet H: Analgesic effects of dexamethasone in burn injury. Reg Anesth.Pain Med. 2002; 27: 254-60

7. Kupers R, Frokjaer VG, Naert A, Christensen R, Budtz-Joergensen E, Kehlet H, Knudsen GM: A PET [18F] altanserin study of 5-HT2A receptor binding in the human brain and responses to painful heat stimulation. Neuroimage. 2009; 44: 1001-7

8. Werner MU, Duun P, Kraemer O, Lassen B, Kehlet H: Arthroscopic Knee Surgery Does Not Modify Hyperalgesic Responses to Heat Injury. Anesthesiology 2003; 99: 1152-7

9. Werner MU, Mjobo HN, Nielsen PR, Rudin A: Prediction of Postoperative Pain: A Systematic Review of Predictive Experimental Pain Studies. Anesthesiology 2010; 112:1494-502

10. Pedersen JL, Kehlet H: Hyperalgesia in a human model of acute inflammatory pain: a methodological study. Pain 1998; 74: 139-51

11. Jensen MT, Petersen KL: Gender differences in pain and secondary hyperalgesia after heat/capsaicin sensitization in healthy volunteers. J.Pain 2006; 7: 211-7

12. Naert AL, Kehlet H, Kupers R: Characterization of a novel model of tonic heat pain stimulation in healthy volunteers. Pain 2008; 138: 163-71

13. Zigmond AS, Snaith RP: The hospital anxiety and depression scale. Acta Psychiatr.Scand. 1983; 67: 361-70

14. Sullivan MJL, Bishop SR, Kivik J: The pain catastrophizing scale: development and validation. Psych Assess 1995; 7: 524-32

15. Dixon WJ: The up-and-down method for small samples. Am Stat Assoc J 1965; 60: 967-78

16. Dixon WJ: Staircase bioassay: the up-and-down method. Neurosci.Biobehav.Rev. 1991; 15: 47-50

17. Altman DG: Practical statistics for medical research., First edition. London, Chapman & Hall, 1991,

18. Matthews JN, Altman DG, Campbell MJ, Royston P: Analysis of serial measurements in medical research [see comments]. BMJ 1990; 300: 230-5

19. Altman DG, Bland JM: Measurement in medicine: the analysis of method comparison studies. The Statistician 1983; 307-17

20. Bland JM, Altman DG: Statistical methods for assessing agreement between two methods of clinical measurement. Lancet 1986; 1: 307-10

21. Bland JM, Altman DG: Measuring agreement in method comparison studies [see comments]. Stat Methods Med Res 1999; 8: 135-60

22. Cohen MR, Cohen RM, Pickar D, Kreger D, McLellan C, Murphy DL: Hormonal effects of high dose naloxone in humans. Neuropeptides 1985; 6: 373-80

23. Cohen MR, Cohen RM, Pickar D, Weingartner H, Murphy DL: High-dose naloxone infusions in normals. Dose-dependent behavioral, hormonal, and physiological responses. Arch.Gen.Psychiatry 1983; 40: 613-9

24. Cohen MR, Cohen RM, Pickar D, Murphy DL: Naloxone reduces food intake in humans. Psychosom.Med. 1985; 47: 132-8

25. Cohen MR, Cohen RM, Pickar D, Weingartner H, Murphy DL, Bunney WE, Jr.: Behavioural effects after high dose naloxone administration to normal volunteers. Lancet 1981; 2: 1110

26. Cohen MR, Cohen RM, Pickar D, Murphy DL, Bunney WE, Jr.: Physiological effects of high dose naloxone administration to normal adults. Life Sci. 1982; 30: 2025-31

Formål:

Formålet er at undersøge om administration af naloxon, en selektiv my-opioid-receptor (MOR) antagonist med centrale og perifere effekter, kan re-introducere sekundære hyperalgesi-arealer efter en førstegrads varmeskade hos frivillige forsøgspersoner.

Endpoints

Primært end point:

Primære effektparametre er sekundære hyperalgesi arealer

Sekundære end points:

Sekundære effektparametre er termale tærskler

- WDT- varme detektiontærskel
- HPT – varme smertetærskel

Mekaniske smertetærskler

- MPT – mekanisk smertetærskel

Psykometriske data

- HADS – Hospital Anxiety and Depression Scale
- PCS – Pain Catastrophizing Scale

Etiske overvejelser

Overvejelser i forbindelse med afprøvningen som helhed:

Vi har udfra gennemgang af 16 studier med høj-dosis naloxon (Bilag 5) vist at der er brugt intravenøse naloxon doser fra 2 – 4 mg/kg hos frivillige og fra 2 – 5 mg/kg hos patienter. Til patienter er der brugt intravenøse naloxon infusioner med indgift av 5 – 70 mg/kg indenfor 1 døgn. De rapporterede bivirkninger dosis-relaterede, uden alvorligere bivirkninger selv hos kritisk syge patienter. Indenfor dosis-intervallet 2 til 4 mg/kg til frivillige forsøgspersoner er de beskrevne bivirkninger af mild intensitet og ser ud til at forekomme hos et mindretal (ca. 30%): kvalme, svaghed, træthed, tremor og øgning af

blodtryk og respirationsfrekvens.

Da forsøgspersonerne screenes inden undersøgelsen med EKG og ultraludsundersøgelse af hjertet (UKG), og under forsøget monitorereres med EKG, pulsoxymetri- og blodtryks-målinger, mener vi på baggrund af litteraturgennemgangen (Bilag 5) ikke at der er specielle etiske problemstillinger i undersøgelsen.

I dette forsøg ønsker vi at undersøge om frivillige forsøgspersoner kan have en ”latent hyperalgesi”, dvs tegn på ”overfølsomhed” af nervesystemet selv lang tid efter en mindre varmeskade. Sådanne fænomener er observeret i dyreeksperimentelle studier og det er vigtigt at undersøge om noget lignende kan ses hos mennesker.

Forsøgspersonen vil ikke direkte drage fordel af dette forsøg, men den viden det forventes at studiet fører med sig vil øge vor forståelse af smertereguleringen hos mennesket og muligvis på sigt kunne føre til bedre smertebehandling.

Undersøgelsen vil blive udført i overensstemmelse med principperne i Helsinki Deklarationen og opdateringer til denne. Protokollen vil blive indsendt til den regionale Videnskabsetiske Komité, Sundhedsstyrelsen samt Datatilsynet til godkendelse. Investigator vil desuden informere den Videnskabsetiske komité, Sundhedsstyrelsen og Datatilsynet om væsentlige eller større ændringer i protokollen.

Risici og ulemper mv.:

Ad. varmeskaden

Den humane varmeskademodel er tidligere anvendt i en række undersøgelser godkendt af Videnskabsetisk Komité (H-1-2009-132, H-2-2010-115, H-4-2011-101, H-2-2012-036). Varmeskaden er sammenlignelig med en lokaliseret let solskoldning (førstegradsforbrænding) og er normalt reversibel inden for 24-48 timer. Smerteintensiteten under skaden er moderat (ifølge vores seneste undersøgelse, H-1-2009-132, VAS [0-100] 50 + 22 [middel, SD]) og risikoen for vedvarende skade er minimal. I en af undersøgelserne (H-1-2009-132) er der dog set vabledannelse med efterfølgende sårdannelse, svarende til en anden grads varmeskade, i 18 tilfælde ud af 300 varmeskader (6 %). Dette er langt højere end tidligere rapporteret, hvor der er set en forekomst på 1 ud af 300 (< 1 %). Dette skyldtes et defekt varme- element, der siden da er udskiftet og erstattet med en sikrere termode type (såkaldt ”parallelt koblede Peltier-elementer” [att. B. Johansson, Somedic Sales AB, Hörby, Sverige]). Hændelsen er rapporteret til og gennemgået af Videnskabsetisk

Komité.

I forbindelse med undersøgelse H-2-2010-115 anskaffede vi et professionelt udstyr for kontaktmålinger af overflade-temperaturer (Testo 735, Lenzkirch, Tyskland). Apparatet har en måleusikkerhed på ± 0,1 ºC.
Vi har foretaget regelmæssige kontroller af termodernes temperatur og i undersøgelserne H-2-2010-115, H-4-2011-101 og H-2-2012-036, hvor der er foretaget mere end 190 varmeskader har vi ikke set vabledannelse hos nogen forsøgsperson. Der vil for at undgå lignende hændelser også i denne undersøgelse blive foretaget kontrolmålinger af varme-elementets temperatur én gang hver 14:e dag.

Ad. lægemiddeltilførsel

Da lægemiddeltilførsel sker intravenøst sættes et perifert i.v.-kateter. Der kan i forbindelse hermed være ubehag relateret til nålestikket. Ved mislykket forsøg til vene-punktion gentages nålestikket, og der kan opstå en mindre blodudtrædning. Disse ubehag anses for at være af lettere karakter.
Indenfor dosis-intervallet 2 til 4 mg/kg til frivillige forsøgspersoner er de beskrevne bivirkninger af mild intensitet og ser ud til at forekomme hos et mindretal (ca. 30%): kvalme, svaghed, træthed, tremor og øgning af blodtryk og respirationsfrekvens (Bilag 5). Forsøgspersonerne screenes inden undersøgelsen med EKG og ultraludsundersøgelse af hjertet (UKG) og under forsøget monitorereres forsøgspersonerne med EKG, pulsoxymetri- og blodtryks-målinger.

Som tidligere nævnt vil undersøgelsen blive udført i overensstemmelse med principperne i Helsinki Deklarationen og opdateringer til denne. Protokollen vil blive indsendt til den regionale Videnskabsetiske Komité, Sundhedsstyrelsen samt Datatilsynet til godkendelse. Investigator vil desuden informere den Videnskabsetiske komité, Sundhedsstyrelsen og Datatilsynet om væsentlige eller større ændringer i protokollen.

Alle forsøgspersoner, der deltager i undersøgelsen, skal give deres mundtlige og skriftlige informerede samtykke, før de inkluderes i undersøgelsen. Det er investigators pligt at informere forsøgspersonerne mundtligt og skriftligt, således at de er fuldstændig klar over

alle aspekter ved deltagelse i undersøgelsen.

Forsøgspersonen, kan på ethvert tidspunkt trække sit samtykke om deltagelse i undersøgelsen tilbage. Hvis forsøgspersonen beslutter at gøre dette, vil det ikke forringe forsøgspersonens forhold til investigator.

Information af og samtykke fra patienter:

De frivillige forsøgspersoner vil blive rekrutteret via fortegnelser over tidligere deltagere med varmeskademodellen (H-2-2010-115, H-4-2011-101, H-2-2012-036) samt ved hjælp af opslag i forsoegsperson.dk og i MOK, de medicin-studerendes blad, på SUND i København. De frivillige forsøgspersoner der henvender sig, vil få tilsendt et kopi af den skriftlige deltager information, og vil blive mundtligt informeret om at tage en bisidder med til den mundtlige information, hvis de ønsker dette. Forsøgspersonerne vil blive mundtligt informeret af forsøgsansvarlig læge i forsøgslaboratoriet. Dette lokale anvendes kun til dette formål, hvorved informationen vil foregå uden unødig forstyrrelser. Der vil blive givet deltagerne den tid, de har behov for og mulighed for at få svar på spørgsmål. Alle spørgsmål vil blive besvaret til forsøgspersonen tilfredsstillende. Den signerede samtykkeerklæring vil være gældende for forsøgspersonens deltagelse. Den frivillige vil få en kopi af den underskrevne samtykkeformular udleveret.

Før de indgår i det kliniske forsøg, skal deltageren have afgivet sit samtykke til at deltage. Information vedrørende forsøget vil blive givet til deltagerne både i mundtligt og skriftlig form. Forklaringer om indhold, omfang og forudsigelige risici og fordele ved det kliniske forsøg, vil blive givet af Investigator i en form, som er forståelig for dem. Den informerede samtykkeformular vil blive indsendt til godkendelse til den regionale Videnskabsetiske Komite. Det godkendte dokument kan blive yderligere opdateret, når ny og vigtig information fremkommer, som kan have indflydelse på deltagerens villighed til at deltage i forsøget. Det informerede samtykke vil indeholde alle de elementer, som er krævet i henhold til dansk lovgivning.

Foruden de standardkrav, som læger i øjeblikket er forpligtet til at tage hensyn til, når de giver information, skal følgende punkter være omfattet:

- en beskrivelse af målene med forsøget og hvordan det vil blive organiseret
- behandlingstypen
- potentielle negative effekter, som kan tillægges forsøgsbehandlingen
- de procedurer, som gennemføres
- retten til at spørge om yderligere information på ethvert tidspunkt
- deltagerens ret til at trække sig ud af det kliniske forsøg på ethvert tidspunkt uden at give forklaring på årsag
- den aflønning, som ydes
- deltagernes forsikringsdækning og relaterede betingelser

For at sikre medicinsk konfidentialitet og databeskyttelse, vil de underskrevne informerede samtykkeformularer blive opbevaret i Investigators forsøgsarkiv. Investigator vil tillade inspektion af formularerne af autoriserede repræsentanter fra Videnskabs Etisk Komite og datatilsynet.
De enkelte deltagere i forsøget skal vide, at deres personlige journaler kan blive gennemgået under konfidentialitet af repræsentanter fra datatilsynet og Videnskabsetisk Komité, Betingelserne for at sikre anonymitet af data og sikkerheden og konfidentialiteten af databasen skal forklares.

Forsøgsdeltagerne skal være klar over, at de kan nægte at gå ind i forsøget, og at de kan trække deres samtykke tilbage på ethvert tidspunkt uden at dette vil have betydning for deres forhold til investigator. Hvis forsøgspersonen selv vælger at trække sig ud af forsøget vil han blive informeret om, at han ikke vil modtage det fulde honorar. Desuden skal forsøgsdeltageren informeres om at de er dækket af den offentlige patientforsikring, og hvis der bliver behov for at klage skal denne informeres om hvor der kan hentes hjælp til dette.

Beskyttelse af data fra patienter:

Alle oplysninger vil blive behandlet fortroligt og ved indberetning af forsøgsresultater vil forsøgsdeltageren være anonyme og de ansvarlige personer for dette forsøg er underlagt tavshedspligt. Forsøget vil blive anmeldt til Datatilsynet.

Investigator vil føre en identifikationsliste over alle forsøgsdeltagere, som har fået tildelt forsøgsnumre. Denne liste vil indeholde fulde navn og CPR-nummer (bilag 1).

Indsamlede data i form af Case Report Form (CRF) og journal oprettet til studiet vil kun blive gjort tilgængelige for inspektion af autoriserede repræsentanter fra relevante myndigheder. Dette vil primært sige i forbindelse med monitorering fra Københavns Universitets Hospitals GCP enhed.

Forsøgspersonerne vil skriftligt blive informeret om, at resultaterne vil blive opbevaret og analyseret i en computer, at alle oplysninger vil blive behandlet fortroligt og ved indberetning af forsøgsresultater vil forsøgspersonerne være anonyme. Samt at den lokale datalovgivning vil blive overholdt.

Forsøgspersonerne vil også skriftligt blive informeret om muligheden for revision fra relevante offentlige myndigheder – Sundhedsstyrelsen, Videnskabsetisk Komité og Datatilsynet, samt at Københavns Universitets Hospitals GCP Enhed også vil have adgang til disse oplysninger.

Projektet vil følge regler for Good Clinical Practice.

Rammer for studiet:

Tidsplan:

Undersøgelsen forventes påbegyndt: 18.03.2013

Undersøgelsen forventes afsluttet: 18.09.2013

Sted for undersøgelsens udførelse:

Anæstesiologisk afd. 4231, HOC,Rigshospitalet.

Undersøgelsen udføres i et roligt, lyst lokale (22-25˚C, relativ luftfugtighed [RH] 20-75 %) med forsøgspersonen i en komfortabel, liggende stilling. Mellem målingerne må forsøgspersonen færdes frit i de tilstødende lokaler.

Studiedesign:

Forsøgstype:	Placebokontrolleret.
Randomisering:	Computergenereret randomiseringsliste, lukkede kuverter.
Blinding:	Dobbeltblind overkrydsning
Deltagere:	Frivillige forsøgspersoner,
Antal:	43 evaluerbare forsøgspersoner

Studieselektion:

Inklusionskriterier:

Forsøgspersonerne skal opfylde alle følgende kriterier for at være egnede til inklusion i undersøgelsen:

- Sund og rask mand
- Alder $\geq$ 20 år og $\leq$ 35 år
- Forsøgspersoner, som har givet deres skriftlige informerede samtykke til at deltage i undersøgelsen efter at have forstået denne.
- Urinprøve uden spor af opioider (morfin,methadon, buprenorphin)
- ASA 1-2
- Body mass index (BMI): 18 < BMI < 30
- Normal ultralydsundersøgelse af hjertet
- Normalt EKG

Eksklusionskriterier:

Forsøgspersoner, der opfylder et eller flere af følgende kriterier, er ikke egnede til inklusion i denne undersøgelse:

- Forsøgspersoner, som ikke kan samarbejde til undersøgelsen.
- Forsøgspersoner, som ikke forstår eller taler dansk
- Deltaget i andet lægemiddelforsøg de seneste 60 dage
- Nervepåvirkning i det ipsilaterale og det kontra-laterale måleområde (læggene), fx. følger efter traume, discusprolaps osv.
- Neurologisk sygdom.
- Psykiatrisk sygdom
- Brug af psykofarmaka
- Alkohol- og/eller medicinmisbrug – efter investigators skøn.
- Kronisk smertetilstand
- Regelmæssigt brug af analgetika (≥ 2 gange per uge)
- Kendt allergi overfor morfin eller morfin-lignende stoffer (inklusive naloxon)
- Hudlæsioner på måleområderne (læggene).
- Indtagelse af receptpligtig medicin 1 uge inden forsøget.
- Indtagelse af håndkøbs medicin op til 48 timer inden forsøget (med undtagelse af antikonceptiva)
- Efter investigators vurdering ikke er egnet til deltagelse i forsøget (eks. psykomotorisk ustabile)
- Allergi over for de i undersøgelsen anvendte stoffer.
- Urinprøve med spor af opioider (morfin,methadon, buprenorphin). Der tages prøve før hver undersøgelsesdag (Dag 0 – 4).

Praktiske forhold:

Økonomiske forhold:

Initiativ til opstart af projektet er taget af professor Jørgen B Dahl og Overlæge Mads Werner.

Udgifter i forbindelse med studiet relaterer sig til diverse anmeldelser, samt forsøgsmedicinen, aflønning af frivillige, aflønning af personale til at hjælpe med projektet, projektsygeplejerske Thomas K.Ringsted.

Forsøgspersonerne vil modtage 175 kr/time for deltagelse i studiet. Det fulde beløb er

skattepligtigt. Alle involverede er ansat på Tværfagligt Smertecenter afsn. 7612 eller HOC, RH. Udgifter bortset fra anmeldelse af studiet afholdes af Professor Jørgen B. Dahls -forskningskonto. Fondskontoen er underlagt offentlig revision.

De forsøgsansvarlige er ansat på Rigshospitalet, og har ingen økonomiske interesser i forsøget.

Forsøgspersonens gennemførelse og afbrydelse af forsøget:

- En forsøgsdeltager, der har gennemført undersøgelsen, er en forsøgsdeltager, der har fulgt forsøgets behandlingsplan i de foreskrevne 168 timer efter varmeskaden.
- En forsøgsdeltager, der ikke har gennemført forsøget, er en forsøgsdeltager, som inkluderes i forsøget, dvs. giver informeret samtykke og ikke gennemfører forsøget, hvad enten forsøgsdeltageren har modtaget forsøgsmedicinering eller ikke.
- Har en forsøgsdeltager ikke gennemført forsøget skal der redegøres for, om eller hvorledes denne følges i øvrigt i studiet, - dette gælder også ”dropouts” - samt hvilke data der indsamles fra disse forsøgspersoner.

Årsager til forsøgspersonens udtrædelse af forsøget:

En forsøgsperson kan tages ud af forsøget under følgende omstændigheder:

- Hvis investigator skønner, at en afbrydelse af forsøget vil være det bedste for forsøgspersonen
- Hvis forsøgspersonen ønsker at udgå af forsøget
- Overtrædelse af protokollens regler

Procedure for patienter, der afbryder forsøget

I overensstemmelse med Helsinki Deklarationen har forsøgspersonerne ret til at afbryde forsøget på ethvert tidspunkt af hvilken som helst årsag. Investigator har også ret til at trække en forsøgsperson ud af forsøget på ethvert tidspunkt.

Årsagen til at en forsøgsperson tages ud af forsøget før planlagt, skal noteres i patientens Case Report Form.

Metodologi:

Generel behandlingsplan og medicindosering

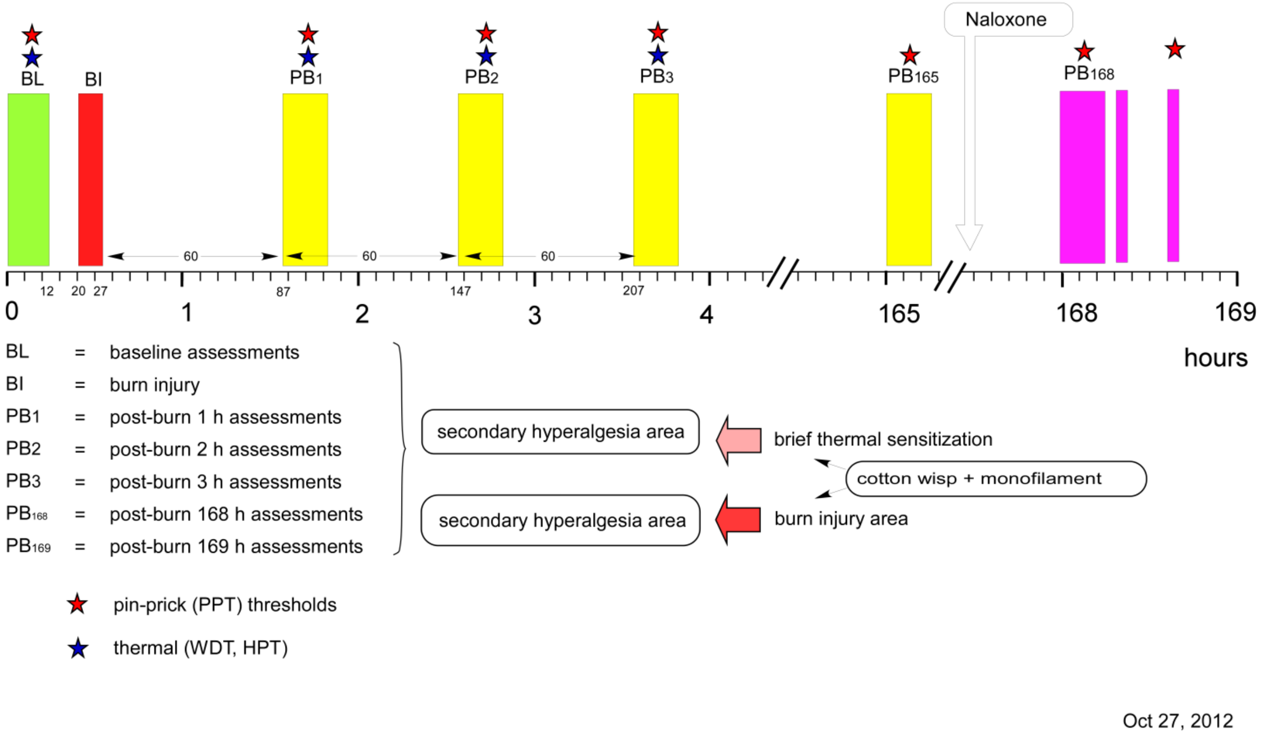

Figur 2. Algoritme over forsøget.

- Det undersøges om patienten opfylder in- og eksklusionskriterierne.
- Forsøgsperioden strækker sig over 5 fremmøder. Dag 0 er forundersøgelses dag. Dag 1 (0-4 timer) og Dag 2 (165-169 timer) er første måle-session, og, Dag 3 (0-4 timer) og Dag 4 (165-169 timer) er anden måle-session. Administration af placebo respektive naloxon sker randomiseret ved første eller anden måleseesion. Der må maximum være 2 uger mellem Dag 0 og Dag 1. Der skal være 168 timer mellem Dag 1 og Dag 2, respektive Dag 3 og Dag 4. Mellem Dag 2 og Dag 3 må der minimum være 3 uger og maximum 4 uger.

    - Dag 0:
        - Indhentelse af informeret samtykke og fuldmagt.

- In- og eksklusionskriterier sikres,
- Der foretages EKG-undersøgelse
- Der foretages ultralydsundersøgelse af hjertet (UKG)
- Udfyldelse af "*Hospital Anxiety and Depression Scale*"
- Udfyldelse af "*Pain Catastrophizing Scale*"
- Objektiv undersøgelse
  - Anamnese
  - Stetoskopi af hjerte og lunger
  - BT og puls
  - Palpation af lymfekirtelstationer
  - Testning af dybe senereflekser
- En første grads varmeskade induceres på det dominante lårs forside og hyperalgesiarealet omkring varmeskaden bestemmes 1 time efter. Tidsforbruget for Dag 0 vil være ca. 3 timer.

o Dag 1/Dag 3:

Forsøgspersoner med hårvækst i måleområdet på den non-dominante læg (Dag 1) og den dominante læg (Dag 3), foretager 2 døgn inden undersøgelsen en skånsom barbering for at undgå påvirkning af de mekaniske målinger.

- Urinprøve testes for opioider
- Randomisering foretages (Dag 1)
- Til tiden 0 foretages baseline målinger (termale tærskler, mekaniske tærskler, "sekundære" hyperalgesi-arealer [i varmeskade området og ved "brief thermal stimulation" (BTS))
- Til tiden 20 minutter induceres en førstegrads varmeskade på forsøgspersonens mediale, proximale del af læggen.
- Til tiden 87, 147 og 207 minutter (1, 2, 3 timer efter varmeskaden) foretages bestemmelser af sekundære hyperalgesiarealer omkring varmeskaden og ved BTS.
- Tidsforbruget for Dag 1/Dag 3 vil være 4-5 timer.

o Dag 2/Dag 4:

- Urinprøve testes for opioider.

- Til tiden 165 timer, 168 timer, 168 timer 15 min og 168 timer 30 min foretages bestemmelser af sekundære hyperalgesiarealer omkring varmeskaden og ved BTS.
- Til tiden 165 timer, 168 timer og 168 timer 30 min foretages bestemmelse pin-prick smerte-tærskler bilateralt.
- Til tiden 165 timer 20 minutter sættes et perifert intravenøst kateter
- Til tiden 167 timer 20 min startes 10 min hurtig intravenøs infusion af naloxon/placebo.
- Tidsforbruget for Dag 2/Dag 4 vil være 4 timer.

Psykometriske skalaer

Dag 0 foretages psykometriske evalueringer, som består af:

- ”*Hospital Anxiety and Depression Scale*” (HADS)[13] til bedømmelse af angst og depression. Ud fra 14 spørgsmål om forsøgspersonens tilstand i den foregående uge, måler HADS via to subskalaer (der hver indeholder syv spørgsmål) hhv. uro/angst og depression. Forsøgspersonen graduerer sine svar på en skala fra 0 til 3. De to subskalaer summeres hver for sig, hvilket maksimalt giver 21 point og en score på 11 eller derover indikerer en mulig forekomst af angst eller depression. Ved en score > 11 point i depressionsdelen af HADS skal en læge gennem en samtale afgøre om der er tale om der er kliniske tegn på depression. Hvis der findes tegn på depression skal mistanken forelægges forsøgspersonen. Hvis forsøgspersonen ønsker det henvises til egen læge for udredning og evt. behandling. Det skal bemærkes at diagnosen depression er en klinisk bedømning hvor en HADS-score eventuelt kan indgå som en del i vurderingen.[1] Forsøgspersonen udgår af studiet hvis der findes kliniske tegn på depression i henhold til exklusionskriterierne.
- ”*Pain Catastrophizing Scale*” (PCS)[14] indeholder 13 spørgsmål som inddeles i tre hovedområder: grublen (rumination), overdrivelse og hjælpeløshed. Spørgsmålene besvares ud fra en skala fra 0 til 4. Ved en total score på $\geq$ 30 er der evidens for katastroficerende tanker.

[1] Cosco TD, Doyle F, Ward M, McGee H: Latent structure of the Hospital Anxiety And Depression Scale: a 10-year systematic review. J.Psychosom.Res. 2012; 72: 180-4

Disse spørgeskemaer anvendes til at afdække forsøgspersonernes smerte-psykologiske profil.

Tærskelbestemmelser.

Den psyko-fysiologiske del af undersøgelsen starter med udmåling og markering med spritpen af måleområdet (2,5 x 5 cm^2) på den mediale, proksimale del af den øvre mediale del af læggen. Kvantitativ sensorisk testning (QST), som bruges ved ”baseline” og de efterfølgende ”post-burn målinger (fig. 2), består af følgende målinger:

- det *sekundære hyperalgesi areal*, der udvikles i den normale hud omkring varmeskaden, undersøges med et stift nylon filament (nominal value 18 [0.89 + 0.05 N (middel + SD)], Stoelting, IL, USA) og med en bomuldspind (ørepind). Grænserne for øget smertefølsomhed bestemmes ved at stimulere langs 8 symmetriske linjer, der indbyrdes danner vinkler på 45°, og som konvergerer ind mod skadens centrum, mens forsøgspersonen holder øjnene lukket. Disse oktagonale arealer overføres efter sessionens afslutning til et transparent overheadark. Arealet beregnes efter indscanning til en JPEG-fil, via et vektorbaseret tegneprogram (Canvas 12.0, ACD systems, USA). De sekundære hyperalgesi arealer bestemmes 0, 1, 2, 3, 165, 168, 168_{15} og 168_{30} timer efter varmeskaden (BL, PB1, PB2, PB3, PB165, PB168, $PB168_{15}$ og $PB168_{30}$ [fig. 2]).
- *termale tærskelbestemmelser* med en 2,5 x 5,0 cm^2 kontakt-termode som appliceres i måleområdet (MSA Thermotest, Somedic AB, Sverige). Der foretages målinger af varmedetektionstærsklen (WDT), og varmesmertetærsklen (HPT) hvor patienten ved hjælp af en trykknap skal afbryde stimuleringen ”lige præcis når temperaturen ændrer sig” respektive ”når varmen bliver til smerte”. Stimulationerne gentages 3 gange med 4-6 s randomiserede intervaller. Termoneutralitet er defineret ved 32 °C og der bruges ved opvarmningen, en ramp rate på 1 °C/s. Hvis patienten ikke trykker på knappen inden 50 °C (WDT, HPT), afbrydes temperaturstimuleringen og termodetemperaturen går tilbage til 32 °C. Termale tærskelbestemmelser gøres ved 0, 1, 2 og 3, timer efter varmeskaden (BL, PB1, PB2, PB3 [fig. 2]).
- *mekaniske smerte-tærskelbestemmelser* (MPT) med en pin-prick stimulator (PinPrick, MRC Systems, Heidelberg) der ved applikation påvirker huden med en 256 mN (kontakt-areal 0,31 mm^2). Der bruges ”down-stroke” og ”up-stroke” hver

med en varighed på 2 sekunder. Der foretages 5 målinger i henhold til Dixon's "op-og-ned"-metode[15;16] i varmeskade-området. Mekaniske smertetærskelbestemmelser gøres ved base-line målinger (fig. 2) og PB165, PB168 og $PB168_{30}$.

Varmeskaden

Efter base-line målingen (fig. 2 BL) påføres forsøgspersonen en overfladisk første grads varmeskade (fig. 2), på medialsiden af den non-dominante læg med en 2,5 x 5 cm^2 kontakttermode (Thermotest, MSA, Somedic AB, Sverige). Termoden appliceres over måleområdet og fastholdes med elastisk bind (Tubegaze). Termoden opvarmes fra 32°C til 47°C som vedligeholdes i 420 sek. Forsøgspersonen kan til hver en tid afbryde varmestimulus ved at trykke på en knap. Smerte intensiteten evaluerer forsøgspersonen med hjælp af en elektronisk VAS-lineal (0-10 [0 = ingen smerte, 10 = værst tænkelige smerte]) 0, 15, 30, 45, 60, 90, 120, 180, 240, 300, 360 og 420 sek efter at termoden har opnået 47°C.

Kort varmestimulation (brief thermal stimulation).

Kort varmestimulation (BTS) sker med termoden (5 x 2,5 cm^2) placeret på forsiden af låret på den dominante side (inferiore kant af termoden placeret 16 cm superiort for overkanten af patella [liggende stilling]). Ved BTS varmes termoden op til 45 ºC i 3 minutter og med termoden in situ undersøges derefter det sekundære hyperalgesi areal i henhold til ovenstående afsnit sekundære hyperalgesi areal. De sekundære hyperalgesi arealer efter BTS undersøges ved baseline og 1 time, 2 timer, 3 timer, 165 timer, 168 timer, 168 timer 15 min og 168 timer 30 min foretages bestemmelser af sekundære hyperalgesiarealer omkring varmeskaden og ved BTS. (fig. 2: BL, PB1, PB2, PB3, PB165, PB168, $PB168_{15}$ og $PB168_{30}$).

Medicin og medicinhåndtering:

Undersøgelsesmedicin:

Aktivt præparat: Naloxon 4 mg/ml, glasampul på 50 ml. Naloxon er opløst 0.9% NaCl opløsning: 1 l af opløsningen indeholder 9 g natriumchlorid i sterilt vand. Elektrolytindhold/l:

154 mmol chlorid og 154 mmol natrium. Isotonisk. Osmolaritet ca. 308 mmol/l.

Dispenseres i ampuller.
Præparatet fremstilles via Skanderborg Sygehus Apotek (att. farmaceut Aage Rasmussen).

Placebo præparat:
Placebo: 0.9% NaCl-opløsning, glasampul på 50 ml. En l af opløsningen indeholder 9 g natriumchlorid i sterilt vand. Elektrolytindhold/l: 154 mmol chlorid og 154 mmol natrium. Isotonisk. Osmolaritet ca. 308 mmol/l.

Blindingsprocedure, pakning og etikettering:
Undersøgelsen er overkrydset, dobbeltblind og randomiseret. Randomiseringen randomization.com vil blive foretaget af Skanderborg Sygehus Apotek. Forsøgsmedicinen vil blive pakket og etiketteret af Skanderborg Sygehus Apotek efter givne regler for dette. Den enkelte patient modtager medicinen som anført under **"Generel behandlingsplan og medicindosering".**

Der udarbejdes endvidere 2 sæt forseglede kodekuverter. Disse indeholder oplysning om, hvilken behandling den enkelte forsøgsperson er randomiseret til. Det ene sæt opbevares af sponsor, det andet opbevares af investigator på Anæstesi- og Operationsklinikken, HOC, Rigshospitalet på et sikkert aflåst sted.
Skanderborg Sygehus Apotek opbevarer den afblindede randomiseringsliste, som først vil blive tilsendt investigator, når dataanalyse er foretaget.

Anden behandling:
Anden medicin, som anses nødvendig for forsøgspersonen, vil blive givet efter investigators skøn.
Administration af al anden medicinsk behandling vil blive registreret på den dertil hørende Case Report Form.

Procedurer ved nødstilfælde:
Investigator vil tilse, at der er procedurer og den fornødne ekspertise til at imødegå en

nødsituation, som kan opstå i løbet af undersøgelsen.
Blindingen må kun brydes, hvis den fortsatte behandling af patienten nødvendiggør kendskab til randomiseringskoden. Hvis kodekuverten åbnes, skal dato og årsag registreres, og kuverten skal signeres af investigator. Dette medfører eksklusion af patienten.

Medicinhåndtering

På den aktuelle dag, for den aktuelle forsøgsperson og i henhold til randomiseringen trækkes 50 ml af opløsningen op i en 50 ml sprøjte (IVAC©) som monteres på en pumpe (IVAC® P7000 sprøjte baseret pumpe). Totalt infunderes volumen sv.t. forsøgspersonens kropsvægt/2 (eks. 75 kg sv.t. infusions volumen 37,5 ml)
Intravenøs administration af naloxon eller placebo påbegyndes tid 167 timer 20 min (fig. 2). Det gives en initial dosis sv.t. 2 ml (4 mg)/30 sekunder, hvorefter forsøgspersonens oplevelse af injektionen samt måling af, respirationsfrekvens, blodtryk og puls gøres over 90 sekunder. Derefter sker administration af resten af bolus-infusion på 10 min. Alle procedurer er dobbeltblinde og identiske for Dag **2** og Dag **4**.

Medicinregnskab:

Ansvarlig investigator vil tilse, at undersøgelsesmedicinen opbevares på et sikkert sted og kun udleveres til deltagere i denne undersøgelse. Investigator vil tilse, at der føres nøje regnskab med den udleverede undersøgelsesmedicin. Investigator gør rede for medicin, som ved et uheld eller på anden måde er bortkommet, samt enhver uoverensstemmelse mellem udleveret og returneret medicin.

Bivirkninger

Bivirkninger/uønskede hændelser (AEs = adverse events):

Bivirkninger defineres som enhver skadelig og uønsket hændelse, tegn eller symptomer, der optræder under deltagelse i undersøgelsen, som er tids relateret til administreringen af undersøgelsesmedicinen, hvad enten denne uønskede hændelse anses for at have forbindelse med undersøgelsesmedicinen eller ej. Alle uønskede hændelser skal noteres i

patientens Case Report Form. Hvis en utilsigtet hændelse opstår mere end 6 timer efter seneste administration af undersøgelsesmedicinen, og der ikke er en tilsyneladende årsagssammenhæng eller forbindelse med undersøgelsesmedicinen, anses denne ikke for en utilsigtet hændelse.

Start og slutdato/tidspunkt, sværhedsgrad og følger efter undersøgelsesstoffet skal noteres for enhver uønsket hændelse. Sværhedsgraden af den uønskede hændelse og sammenhængen med undersøgelsesstoffet skal vurderes i overensstemmelse med nedenfor beskrevne retningslinier.

Investigator skal vurdere sammenhæng mellem en uønsket hændelse og undersøgelsesstoffet ved hjælp af følgende retningslinier:

Retningslinier for uønskede hændelsers eventuelle sammenhæng med behandling:

1. Ikke relateret - ingen tidsmæssig sammenhæng, andre ætiologier meget sandsynligt årsagen
2. Muligvis relateret - mindre klar sammenhæng, andre ætiologier er også mulige
3. Sandsynligvis relateret - klar tidsmæssig sammenhæng med bedring ved afbrydelse af medicinering, og ikke rimeligt forklaret ved patientens kendte kliniske tilstand.
4. Relateret - klar tidsmæssig sammenhæng med genbehandlingstest eller klinisk vurdering.

Forsøgspersoner, der har uønskede hændelser, vil blive monitoreret med relevante kliniske vurderinger og laboratorieundersøgelser efter behandlende læges beslutning. Alle uønskede hændelser vil blive fulgt til tilfredsstillende restituering eller stabilisering.

Ved en alvorlig uønsket hændelse forstås en hændelse uanset dosis som medfører en betydelig risiko for død eller handikap hos forsøgspersonen inkluderende, men ikke begrænsende sig til, en hændelse som resulterer i:

- død
- er livstruende – patienten var efter investigators skøn i umiddelbar risiko for at dø af den uønskede hændelse, da den optrådte
- medfører hospitalsindlæggelse eller forlænger allerede eksisterende indlæggelser
- er varigt invaliderende

Der vil blive anvendt produktresume for Naloxon fra Sundhedsstyrelsens hjemmeside,

som referencedokument, når der skal vurderes om en alvorlig relateret bivirkning er ventet eller uventet.

Gradering af uønskede hændelser:

Den lægelige investigator skal forsøge at finde frem til alle kliniske og objektive reaktioner fra patienter i behandling og fastslå deres sammenhæng med undersøgelsesstoffet. Reaktioner, hvis der er nogen, skal graderes efter følgende skala:

1 = let

2 = moderat

3 = svær

4 = livstruende

Rapportering af AEs og SAEs bivirkninger:

Investigator er ansvarlig for at alle uønskede hændelser skal registreres i patientens Case Report Form.

Sponsor er ansvarlig for den løbende overvågning af forsøgets risk/benefit forhold. Opstår eller erkendes der situationer, der kan have betydning for forsøgspersonernes sikkerhed eller forsøgets udførelse skal dette **altid** straks rapporteres til Sundhedsstyrelsen. Tilsvarende rapportering skal også ske til alle involverede investigatorer og Videnskabsetisk Komité.

Herudover gælder nedenstående regler for indberetning til myndigheder. Der skal indberettes hændelser og bivirkninger for al forsøgsmedicin - både den eksperimentelle behandling, comparatorer og evt. placebo.

Sponsor skal af investigator løbende holdes orienteret om adverse events. Ved forsøgets afslutning skal den endelige rapport indeholde en beskrivelse af alle indtrufne bivirkninger.

Serious Adverse Events (SAE) skal omgående indberettes af investigator til sponsor. SAE skal af investigator indberettes én gang årligt til Videnskabsetisk Komité i hele forsøgsperioden, sammen med en rapport over forsøgspersonernes sikkerhed.

Serious adverse reaction (SAR) (Alvorlige, formodede bivirkninger), Skal af sponsor

indberettes én gang årligt til Sundhedsstyrelsen. Indberetningen skal også indeholde en rapport over forsøgspersoners sikkerhed.

Suspected unexpected serious adverse reaction (SUSAR) (Uventede og alvorlige formodede bivirkninger), skal investigator **omgående** orientere sponsor om. Sundhedsstyrelsens produktresume for Naloxon anvendes som referencedokument, ved vurdering af om en alvorlig relateret bivirkning er uventet eller ventet.

Dødelige eller livstruende SUSAR´s indberettes senest **7 dage** efter, at sponsor har fået kendskab til dem, og senest **8 dage** efter indberetningen skal sponsor meddele Sundhedsstyrelsen alle relevante oplysninger om opfølgningen.

Alle andre uventede og alvorlige formodede bivirkninger, skal indberettes til samme myndigheder senest 15 dage efter, at sponsor har fået kendskab til disse.

Enhver indberetning skal ledsages af kommentarer om evt. konsekvenser for forsøget.

Det anbefales endvidere, at sponsor orienterer lægemidlets fremstiller.

Statistiske analyser

Beregning af antal patienter:

Styrkeberegningen hviler på forudsætningerne:[17]

- at data er normalfordelt
- at alpha sættes til 0,01
- at beta sættes til 0,1 (power = 0,9)
- at designet er et crossover studie
- at den estimerede intra-individuelle standard deviation (SD) af sekundære hyperalgesi-arealer 168 timer efter varmeskaden er 3 cm^2 at den minimale relevante difference sættes til 3 $cm.^2$
- at der foretages en interimsanalyse med partiel afblinding (gruppe A og B) af uafhængig statistiker, når halvdelen af forsøgspersonerne er inkluderede.

Antallet af forsøgspersoner der skal inkluderes er således 34.

Da høj-dosis naloxon tilførsel ikke er prøvet ved humane forsøg i Danmark vil vi bruge de 3 første forsøgspersoner som ublindede testpiloter hvor vi bedømmer de subjektive virkninger af høj-dosis naloxon. De 6 næste forsøgspersoner fungerer som test-piloter med henblik på at evaluere bivirkninger og farmakodynamisk effekt (genindsættelse af sekundære hyperalgesi arealer). Det indebærer at disse 6 forsøgspersoner følger prokollen, hvorefter vi afblinder data i en A- og B-gruppe (delvis afblindning) og sammenstiller disse data. Derefter foretages fuld afblindning. Sponsor og investigator diskuterer derefter om undersøgelsen skal fortsætte. Dette hviler på graden af bivirkninger og på om den ønskede effekt på sekundære hyperalgesi-arealer er tilstede. Data fra disse 6 forsøgspersoner indgår ikke i den samlede undersøgelse. Det totale antal forsøgspersoner er derfor 3 + 6 + 34 = 43 evaluerbare. Det maksimale antal forsøgspersoner der søges om tilladelse er 49.
Interimsanalysen er begrundet i undersøgelsens hypotese-genererende status: der findes ikke humane data der kan bruges ved styrkeberegningen. Interimsanalysen bruges udelukkende til at checke om de statistiske *a prori* forudsætninger holder, d.v.s. om antallet af forsøgspersoner er tilstrækkeligt for at bevise en statistisk forskel mellem grupperne. Er forudsætningerne holdbare fortsættes undersøgelsen. Er forudsætningerne ikke holdbare diskuterer sponsor og investigator om der skal ansøges om tilladelse til at inkludere et større antal forsøgspersoner.

Fordelingen af kontinuerlige data bedømmes for normalitet med residual-plots eller Shapiro-Wilk W test (SPSS 20.0, Chicago, IL, USA). Normalitet kan forsøges opnået med simpel transformation (log, logit). Statistisk sammenligning af parrede data gøres med parametriske eller non-parametriske metoder, afhængig af fordelingen, med Mann-Whitney, respektive Wilcoxon test. Risikoen for type I fejl er reduceret ved at signifikansnivauet er sat til 0,01 (alpha). Der bruges generelt summations mål,[18] for at undgå massesignifikans ved gentagne målinger, og der endvidere brugt en ”power” på 0,9 (beta = 0,1) for yderligere at mindske risikoen for type II fejl. Ved statistiske beregninger, hvor multiple sammenligninger ikke kan undgås, bruges Duncan´s korrektion (ex. sekundære outcome parametre). Ved statistiske sammenligninger mellem kategori variabler (eks. psykometriske data) bruges $\chi 2$-test med Yates’ korrektion.

Databearbejdning:

Dataregistrering, samt regler for kontrol af undersøgelsesprocedurer:

Undersøgelsen skal gennemføres i overensstemmelse med de gældende regler for kliniske forsøg, der omfatter mennesker vedrørende kvalitetskontrol og kvalitetsstyring, og vil følge Good Clinical Practice - guidelines.

Investigator er tilknyttet Rigshospitalet og er ansvarlige for håndtering og arkivering af data efter gældende regler herunder lov om behandling af personoplysninger og sundhedsloven.

Data tilhører sponsor.

Projektet er anmeldt til datatilsynet.

Case Report Form:

For hver patient inkluderet i undersøgelsen vil en Case Report Form (CRF) blive udfyldt. Denne vil blive signeret af investigator for at bekræfte rigtigheden af data. Rettelser af data vil kun blive foretaget ved at strege de forkerte data ud (den ukorrekte information vil forblive synlig og læselig) og de korrekte data vil blive skrevet ved siden af de udstregede. Korrektionslak vil ikke blive anvendt. Rettelser vil blive dateret og signeret af investigator eller dennes stedfortræder. Case Report Form er vedlagt som bilag 4.

Sourcedata er journal og CRF.

Monitorering:

Københavns Universitets Hospitals GCP Enhed
Bispebjerg Hospital, Bygning 51, 3. sal
Bispebjerg Bakke 23
2400 København NV
Tlf: 3531 3890

Uddannelse:

Forskningssygeplejerske Thomas Ringsted vil sikre, at det involverede personale er

passende uddannet og instrueret og har de fornødne oplysninger til udførelse af undersøgelsen.

Yderligere krav og generel information:

Forsikring:

For frivillige forsøgspersoner er gældende at med hensyn til enhver skade, forårsaget direkte eller indirekte af undersøgelsesmedicinen i denne kliniske undersøgelse, påtager Tværfagligt Smertecenter, afsnit 7612, Neurocentret, Rigshospitalet, sig det lovmæssige ansvar på investigator og dennes medarbejderes vegne, forudsat at investigator og dennes medarbejdere har fulgt de instruktioner, som er givet i denne protokol samt eventuelle tillæg dertil, samt at investigator og dennes medarbejdere har udført undersøgelsen videnskabeligt og i overensstemmelse med gældende regler og accepterede teknikker. Forsøgspersoner er i tilfælde af skade eller død uden sammenhæng med undersøgelsens gennemførelse forsikret af hospitalets forsikring.

Offentliggørelse af resultater:

På basis af data vil investigator skrive en rapport over undersøgelsen. Denne rapport vil blive fremsendt til relevante myndigheder. Rapporten vil også danne basis for et manuskript til publikation med følgende forfatterrækkefølge:

Pereira MP, Ringsted TK, Bischoff JM, Taylor B, Dahl JB*, Werner MU
* korresponderende forfatter.

Såvel negative, positive og inkonklusive forsøgsresultater vil blive offentliggjort.
